# Environment and Health in Contaminated Sites: The Case of Taranto, Italy

**DOI:** 10.1155/2013/753719

**Published:** 2013-12-24

**Authors:** Roberta Pirastu, Pietro Comba, Ivano Iavarone, Amerigo Zona, Susanna Conti, Giada Minelli, Valerio Manno, Antonia Mincuzzi, Sante Minerba, Francesco Forastiere, Francesca Mataloni, Annibale Biggeri

**Affiliations:** ^1^Department of Biology and Biotechnologies Charles Darwin, Sapienza Rome University, Piazzale Aldo Moro 5, 00185 Rome, Italy; ^2^Department of Environment and Primary Prevention, National Health Institute, Viale Regina Elena 299, 00161 Rome, Italy; ^3^Unit of Statistics of the National Health Institute, National Center for Epidemiology, Surveillance and Health Promotion, Viale Regina Elena 299, 00161 Rome, Italy; ^4^Taranto Local Health Unit, Epidemiological and Statistical Unit, Viale Virgilio 31, 74121 Taranto, Italy; ^5^Department of Epidemiology, Lazio Regional Health Service, Via di Santa Costanza 53, 00198 Rome, Italy; ^6^Biostatistics Unit, ISPO Cancer Research and Prevention Institute, Via Cosimo il Vecchio 2, 50139 Florence, Italy; ^7^Annibale Biggeri Department of Statistics “G. Parenti”, University of Florence, Viale Morgagni 59, 50134 Firenze, Italy

## Abstract

The National Environmental Remediation programme in Italy includes sites with documented contamination and associated potential health impacts (National Priority Contaminated Sites—NPCSs). SENTIERI Project, an extensive investigation of mortality in 44 NPCSs, considered the area of Taranto, a NPCS where a number of polluting sources are present. Health indicators available at municipality level were analyzed, that is, mortality (2003–2009), mortality time trend (1980–2008), and cancer incidence (2006-2007). In addition, the cohort of individuals living in the area was followed up to evaluate mortality (1998–2008) and morbidity (1998–2010) by district of residence. The results of the study consistently showed excess risks for a number of causes of death in both genders, among them: all causes, all cancers, lung cancer, and cardiovascular and respiratory diseases, both acute and chronic. An increased infant mortality was also observed from the time trends analysis. Mortality/morbidity excesses were detected in residents living in districts near the industrial area, for several disorders including cancer, cardiovascular, and respiratory diseases. These coherent findings from different epidemiological approaches corroborate the need to promptly proceed with environmental cleanup interventions. Most diseases showing an increase in Taranto NPCS have a multifactorial etiology, and preventive measures of proven efficacy (e.g., smoking cessation and cardiovascular risk reduction programs, breast cancer screening) should be planned. The study results and public health actions are to be communicated objectively and transparently so that a climate of confidence and trust between citizens and public institutions is maintained.

## 1. Introduction

Contaminated sites are extensively present in Europe where approximately 250,000 sites require cleanup interventions, as listed by the European Environment Agency [1]. Several thousands of these sites are located in Italy, and a total of 57 sites, defined in 2009 as National Priority Contaminated Sites (NPCSs), qualify for remediation because of contamination documented in qualitative and/or quantitative terms, and because of a potential health impact.

The site of Taranto, located in Apulia region (southern Italy), includes two municipalities and 216,618 inhabitants at 2001 Census. This site is of interest because of several polluting sources, such as a large steel plant, a refinery, the harbor, and both controlled and illegal waste dumps.

Previous environmental and epidemiological investigations in the area have provided evidence of environmental contamination [2–9]. These studies have documented severe air pollution originating mainly from the steel industry, that is, particulate matter, heavy metals, polycyclic aromatic hydrocarbons, and organ-halogenated compounds.

Epidemiological studies showed an increased mortality/morbidity from respiratory, cardiovascular diseases and several cancer sites [10–13].

SENTIERI Project (epidemiological study of residents in National Priority Contaminated Sites—NPCSs) funded by the Italian Ministry of Health studied mortality for 63 causes among residents of 44 NPCSs included in the “National Environmental Remediation Programme” [14].

The distinguishing feature of SENTIERI Project is that the epidemiological evidence evaluation has been carried out before the study to minimize the risk for researchers to be data-driven when performing and interpreting the specific epidemiologic investigation. SENTIERI dealt with the complexity of the relation between area contamination and health effects by examining, for each combination of causes of death/*environmental exposures*, the epidemiological evidence (1998–2009) and then building a matrix of the *a priori* evaluation of the strength of the causal association. The *environmental exposures* were classified as chemicals, petrochemicals and refineries, steel plants, power plants, mines and/or quarries, harbour areas, asbestos or other mineral fibers, landfills, and incinerators—labelled on the basis of the legislative decrees defining the sites' boundaries. A standardized procedure was set up to collect the available epidemiological literature, which was reviewed on the basis of explicit criteria and led to classify each cause of death/*environmental exposures* combination in terms of strength of causal association. The evaluation was categorized as sufficient to infer the presence of a causal association (S), limited to infer the presence of a causal association (L), and inadequate to infer the presence or the absence of a causal association (I). The rationale, scope, methods, and details of the *a priori* evidence evaluation can be found in [15], and the procedures and results of the evidence evaluation have been published in Italian [16].

With specific reference to the *environmental exposures* in Taranto NPCS, this procedure led to classify the presence of a steel industry, a refinery, a harbour area, and a number of landfills and waste dump sites as associated, with limited evidence, with an increased risk of lung cancer, pleural mesothelioma, nonmalignant respiratory diseases, congenital malformations, and perinatal conditions. In this context, residence near steel industry was evaluated as specifically associated with the occurrence of both acute and chronic respiratory diseases in adults and children, based on studies by [17–23]. It should be underlined, however, that air pollution from particulate matter has been causally linked by WHO with several health effects, including all-cause mortality and cardiovascular and respiratory morbidity [24].

SENTIERI analyzed mortality at municipality level in the period 1995–2002, computing standardized mortality ratios (SMR) both crude and adjusted for a deprivation index [25]. While SENTIERI strengths are the *a priori *epidemiological evidence evaluation and the mortality analysis of all NPCSs adopting the same analytical approach and adjusting for deprivation, there are several limitations that should be noted, such as its ecological design and the use of mortality data at municipal level for a short period of time.

With the aim of overcoming the above limitations, this paper presents an epidemiological profile of Taranto NPCS residents analyzing different health indicators available at municipality level, that is, cause-specific mortality (2003–2009), mortality time trend (1980–2008), and cancer incidence (2006-2007). A cohort study of the resident population examined mortality (1998–2008) and morbidity (1998–2010) in the districts close to the steel plant.

## 2. Material and Methods

### 2.1. Data Source

Details on the codes used during the study period for the 9th and 10th revisions of the International Classification of Diseases (ICD-9 and ICD-10) and on the demographic data of the two municipalities included in Taranto NPCS are presented in Appendix A.

### 2.2. Mortality 2003–2009

Mortality in Taranto NPCS residents was initially studied for the period 1995–2002 [12] and then updated for the years 2003–2009 (note that the period 2004-2005 was not available from ISTAT). The analysis considered 63 single or grouped causes (all ages, both genders); 0-1 and 0–14 age classes were also analyzed for a selection of causes (both genders combined). Standardized mortality ratios both crude (SMRs) and adjusted for deprivation together with 90% confidence intervals (90% CIs) were computed using regional rates for comparison [14]. In SENTIERI Project the deprivation index (DI) was constructed using the 2001 national census variables representing the following socioeconomic domains: education, unemployment, dwelling ownership, and overcrowding. The strengths and weaknesses of SENTIERI ID, its correlation with 2001 national deprivation index, its efficacy in representing deprivation in different categories of demographic dimensions, together with suggestions about the use of socioeconomic indices in small area studies of environment and health are discussed in Pasetto et al. 2011 [26].

### 2.3. Mortality Time Trend 1980–2008

Mortality was analyzed for a twenty-seven-year period: 1980–2008. The analysis was performed for the population 0–99 years separately for men and women; directly standardized death rates (SDRs) per 100,000 were calculated (standard: Italian population at 2001 Census) together with their 90% CI. The population size of Taranto NPCS is relatively small; therefore the study period was divided into three-year periods to obtain stable values of the indicators. The overall mortality was analyzed together with specific diseases, selected on the basis of the *a priori* evidence evaluation of their link with *environmental exposures* in Taranto NPCS. The selected diseases were all cancers (in particular lung cancer), circulatory diseases (in particular ischemic heart disease), and respiratory diseases (in particular, the acute, and chronic ones).

We also analyzed the overall infant mortality (i.e. mortality from all causes during the first year of life) without gender distinction, as not informative in this age group.

### 2.4. Cancer Incidence 2006-2007

For cancer incidence (2006-2007), standardized incidence ratio (SIR) and 90% CI were calculated for both genders; the incidence rates of Italian South and Islands Cancer Registries macroarea (2005–2007) and of Taranto Province, excluding NPCSs municipalities (2006-2007), were used for comparison.

### 2.5. Mortality (1998–2008) and Morbidity (1998–2010) of the Residential Cohort

A cohort study design was applied to evaluate cause-specific mortality and hospitalization in relation to residence in specific districts close to the industrial sites. A cohort of residents (all subjects living in Taranto, Massafra, and Statte as January 1, 1998, and subsequently entered in these municipalities up to 2010) was enrolled from the municipal register. Individual follow-up for vital status assessment at 31.01.2010 was performed using municipality data. This cohort population is different from the base population analyzed for mortality in 2003–2009 (see previous paragraph for details). The socioeconomic position level (SEP) of the census block of residence and the district of residence were assigned to each participant (five categories from low to high SEP), on the basis of the addresses geocoded at the beginning of the follow-up. Occupational history for all cohort members was traced through the national insurance company (INPS) database (people employed in 1974 and subsequently), and the subcohort of individuals employed in industries located in the area was identified. Mortality/morbidity information was retrieved from Regional Health Databases (1998–2008 for mortality, 1998–2010 for hospital admissions). The associations of district of residence with mortality/morbidity were estimated by calculating mortality and morbidity hazard ratios (HR, CI 95%) using the proportional Cox models. All models considered age (temporal axis), calendar period, and area-based socioeconomic status [25], and they were calculated separately for men and women [13].

## 3. Results

### 3.1. Mortality 2003–2009-SENTIERI

Tables 1–3 show mortality results in the periods 1995–2002 and 2003–2009.

In Table 1, mortality, from the main causes of death, is displayed for descriptive purposes; Tables 2 and 3 present the results for the causes selected on the basis of the *a priori* evidence evaluation, the distinguishing feature of SENTIERI Project [15].

Table 1 shows that in both periods, for both genders, for all causes and all neoplasms, there was an excess of mortality ranging between 7% and 15%; adjustment for deprivation did not substantially change SMRs values. In both periods, among males and females, the observed mortality was above expected for circulatory, respiratory, and digestive systems diseases; also in these cases, accounting for socioeconomic factors did not essentially change the study results. For diseases of the genitourinary system, the observed mortality was similar to the expected one.

Table 2 presents the results for the causes of death for which SENTIERI classified the epidemiological evidence of causal association with the *environmental exposures* in Taranto NPCS as “Limited”. From now on, reference will only be made to results adjusted for deprivation. Among males, lung cancer showed a 20% excess in the first period, confirmed in the second one; among females the excesses were, respectively, about 30% (1995–2002) and 20%. Correspondingly, in the two periods excesses for pleural tumors were 193% and 167% among males, 90% and 103% among females. Excesses for acute respiratory diseases among males were 49% (1995–2002) and 37% (2003–2009), and for females 38% and 14%, respectively. The observed mortality for chronic respiratory diseases in 1995–2002 was as expected in both genders, while in 2003–2009 a 10% excess was present for males. Asthma mortality was not increased, but the observed number of death is small.

Table 3 displays the results combined for males and females, again for causes with limited epidemiological evidence of causal association with the *environmental exposures* in Taranto NPCS. The mortality from congenital anomalies showed a 17% excess in 1995–2002, while in 2003–2009 it was below expectation. For mortality from perinatal conditions, an heterogeneous group of diseases affecting fetus or newborn spanning from pregnancy and delivery complications to digestive or hematological disorders, the excess was 21% and 47%, in the first and second periods, respectively. In the age class 0–14 less than 3 deaths from acute respiratory diseases and asthma were observed.

Some noteworthy results should be considered (results not shown in Tables). Among males, the observed deaths were above expected in 1995–2002 and 2003–2009 for dementia (resp., 105 and 102 deaths), hypertensive diseases (resp., 307 and 287 deaths), ischemic heart diseases (resp., 1032 and 679 deaths), and cirrhosis (resp., 266 and 156 deaths). In 2003–2009 excesses were reported for melanoma (50%, 26 deaths), non-Hodgkin lymphoma (34%, 45 deaths), and myeloid leukemia (35%, 37 deaths).

### 3.2. Mortality Time Trend Analysis 1980–2008

Time trends for mortality from all causes and selected causes among adults and overall infant mortality are presented in Figures 1–17 and in Appendix B (see Tables S2, S3, and S4 in Supplementary Material available online at http://dx.doi.org/10.1155/2013/753719).

#### 3.2.1. Men

**Figure 1:**
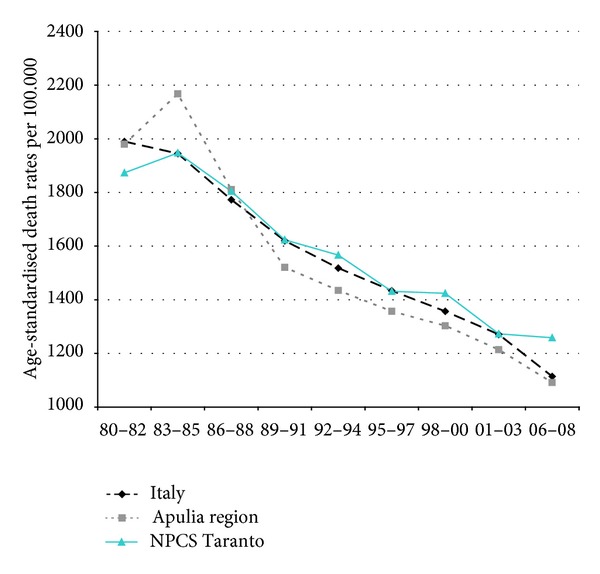
Overall mortality. Trends in age-standardised (Italian Census 2001) death rates per 100.000 (1980–2008) (2004-2005 data were not available). All ages. Men.

Since many decades, overall mortality in Italy and in Apulia has been declining (resp., 44% and 45%). This favorable trend was also observed in Taranto NPCS, where the regular fall showed a slackening in the last three-year period. Since the early 90s, the SDRs observed in Taranto NPCS were higher than those observed in Apulia, which in turn were lower than the Italian ones; in the most recent three-year period the SDR observed in Taranto site was higher than that in Apulia and Italy (Figure 1).

**Figure 2:**
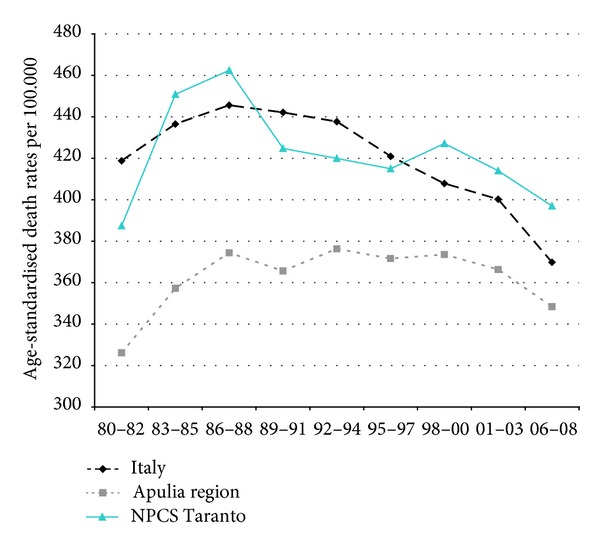
All cancers. Trends in age-standardised (Italian Census 2001) death rates per 100.000, from selected causes of death (1980–2008) (2004-2005 data were not available). All ages. Men.

In Italy, mortality from all neoplasms has been falling throughout the study period, while in Taranto NPCS and in Apulia, the trend has been moderately increasing for the same study period. In Taranto NPCS SDRs tend to be higher than in Apulia, which in turn are lower than in Italy (Figure 2).

**Figure 3:**
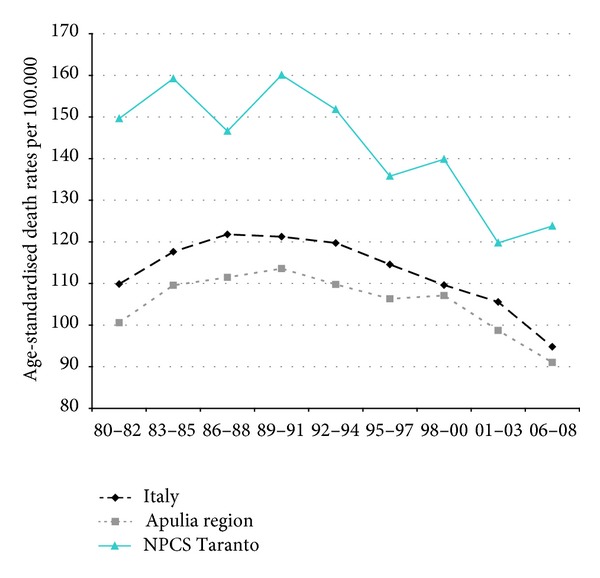
Lung cancer. Trends in age-standardised (Italian Census 2001) death rates per 100.000, from selected causes of death (1980–2008) (2004-2005 data were not available). All ages. Men.

Mortality from lung cancer has been declining in Italy, Apulia, and Taranto NPCS throughout the years 1980–2008; SDRs in the study site are higher than in Italy and Apulia, since 1995–1997, this differential shows a reduction (Figure 3).

**Figure 4:**
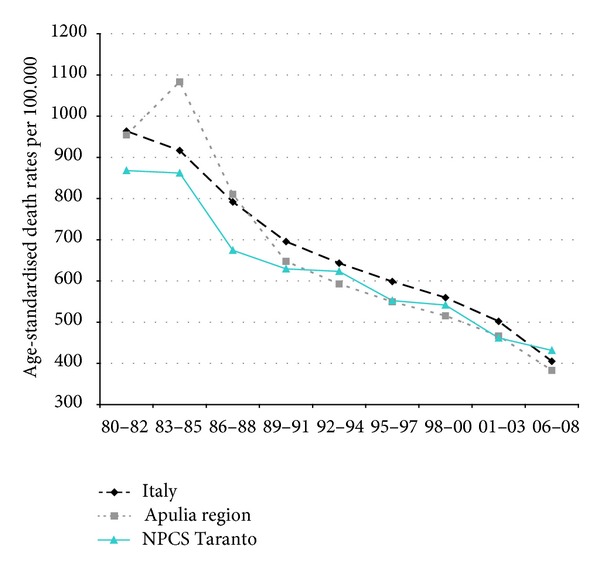
Circulatory diseases. Trends in age-standardised (Italian Census 2001) death rates per 100.000, from selected causes of death (1980–2008) (2004-2005 data were not available). All ages. Men.

Mortality from circulatory diseases in Italy, Apulia, and Taranto site decreased, and the mortality rates almost halved during the study period; yet, in the most recent time, Taranto's mortality rates are higher than in Italy (Figure 4).

**Figure 5:**
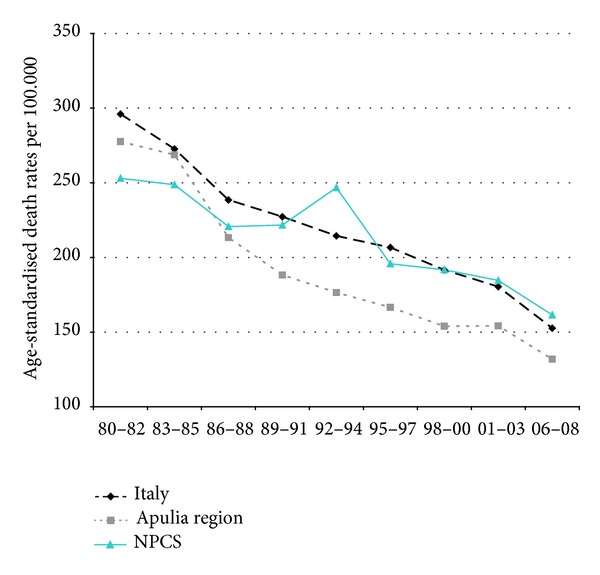
Ischaemic heart diseases. Trends in age-standardised (Italian Census 2001) death rates per 100.000, from selected causes of death (1980–2008) (2004-2005 data were not available). All ages. Men.

Mortality trends from ischemic heart disease have been declining in Italy, Apulia, and Taranto site; yet, since the end of the 1980s, Taranto's SDRs are higher than in Apulia (Figure 5).

**Figure 6:**
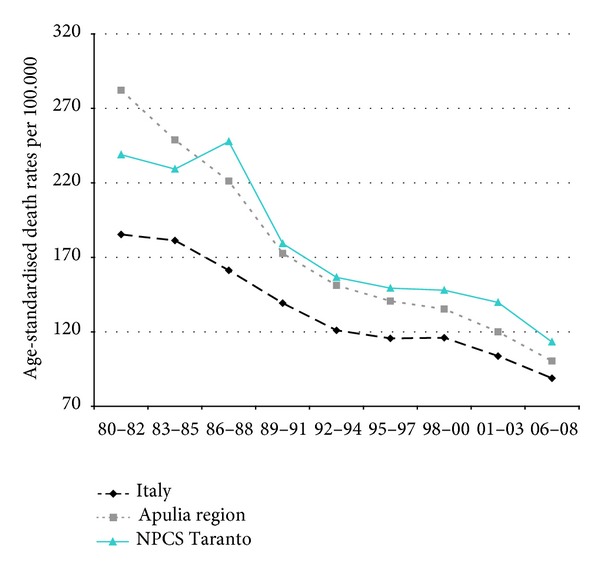
Respiratory diseases. Trends in age-standardised (Italian Census 2001) death rates per 100.000, from selected causes of death (1980–2008) (2004-2005 data were not available). All ages. Men.

**Figure 7:**
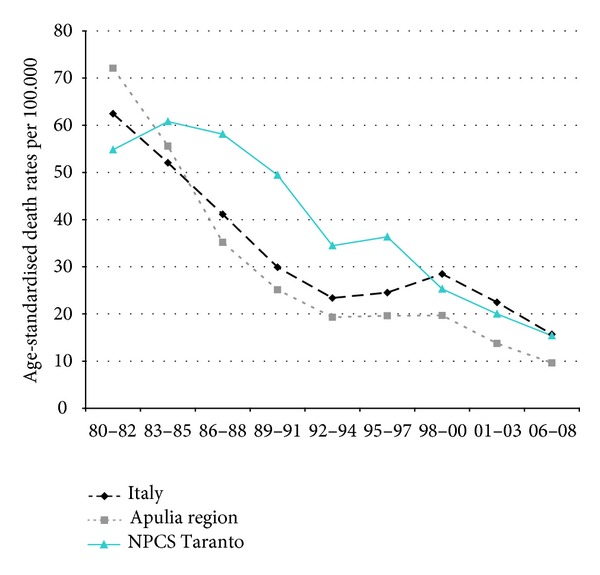
Acute respiratory diseases. Trends in age-standardised (Italian Census 2001) death rates per 100.000, from selected causes of death (1980–2008) (2004-2005 data were not available). All ages. Men.

**Figure 8:**
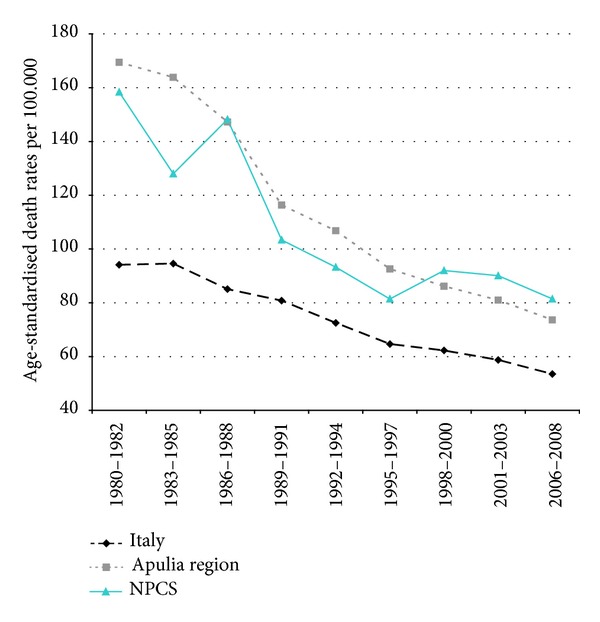
Chronic respiratory diseases. Trends in age-standardised (Italian Census 2001) death rates per 100.000, from selected causes of death (1980–2008) (2004-2005 data were not available). All ages. Men.

Mortality trends from respiratory diseases (overall, acute, and chronic) showed a decrease in Italy, Apulia, and Taranto. The rates in Taranto site are higher than those in the Italian ones over the whole study period, the exception being the last decade, when only mortality due to acute respiratory was higher in Taranto (Figures 6, 7, and 8).

#### 3.2.2. Women

**Figure 9:**
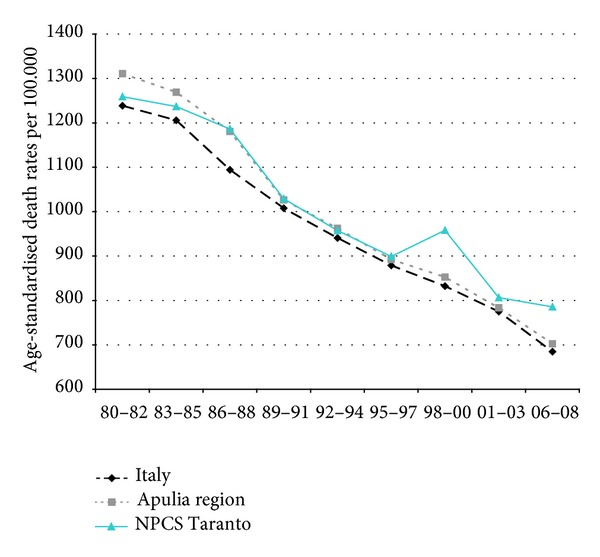
Overall mortality. Trends in age-standardised (Italian Census 2001) death rates per 100.000 (1980–2008) (2004-2005 data were not available). All ages. Women.

Overall mortality among women in Italy, Apulia, and Taranto site showed a long-term decreasing trend: from 1980 to 2008 the decrease was respectively 45%, 46%, and 38%; yet, since the beginning of the 2000s, mortality rates in Taranto are higher compared to those in Apulia and Italy (Figure 9).

**Figure 10:**
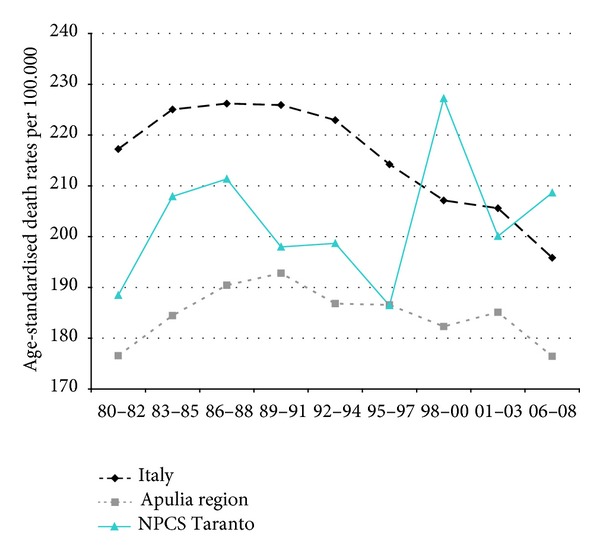
All cancer. Trends in age-standardised (Italian Census 2001) death rates per 100.000, from selected causes of death (1980–2008) (2004-2005 data were not available). All ages. Women.

Mortality from cancer (all sites) has been decreasing in Italy, remaining stable in Apulia, and increasing in Taranto site (Figure 10).

**Figure 11:**
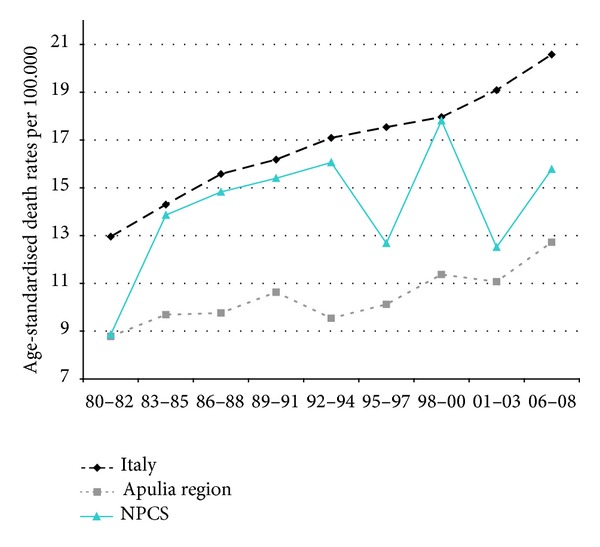
Lung cancers. Trends in age-standardised (Italian Census 2001) death rates per 100.000, from selected causes of death (1980–2008) (2004-2005 data were not available). All ages. Women.

In contrast with the overall cancer mortality, lung cancer mortality has been rising steadily; in the study period the increase was 59% in Italy, 44% in Apulia, and 78% in Taranto NPCS, where SDRs were higher than in Apulia (Figure 11).

**Figure 12:**
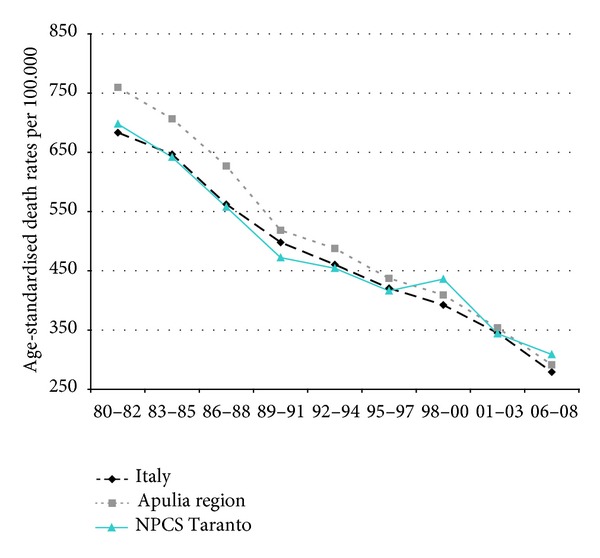
Circulatory diseases. Trends in age-standardised (Italian Census 2001) death rates per 100.000, from selected causes of death (1980–2008) (2004-2005 data were not available). All ages. Women.

**Figure 13:**
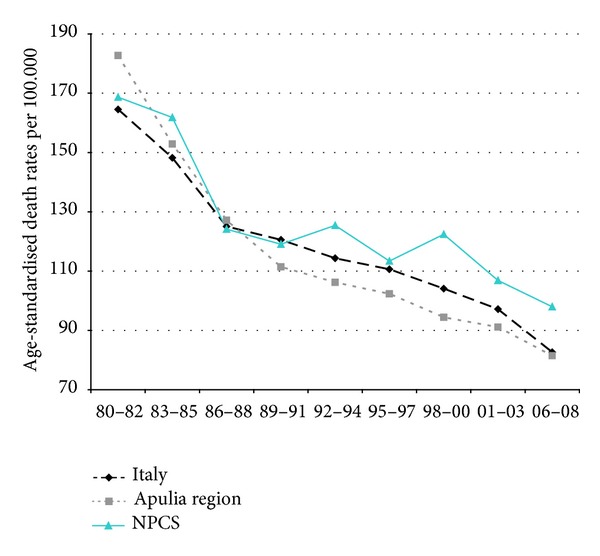
Ischaemic heart diseases. Trends in age-standardised (Italian Census 2001) death rates per 100.000, from selected causes of death (1980–2008) (2004-2005 data were not available). All ages. Women.

Mortality from circulatory diseases showed a declining trend in Italy, Apulia, and Taranto site (Figure 12). Mortality from ischemic heart disease declined as well, but the rates observed in Taranto NPCS were higher than in Apulia and Italy throughout the study period (Figure 13).

**Figure 14:**
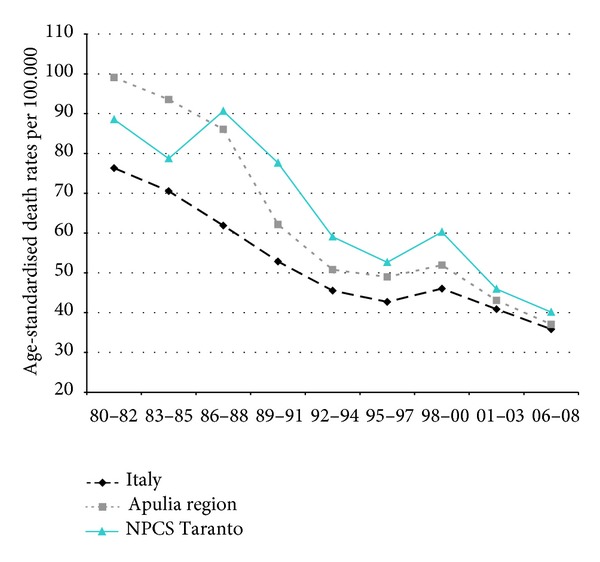
Respiratory diseases. Trends in age-standardised (Italian Census 2001) death rates per 100.000, from selected causes of death (1980–2008) (2004-2005 data were not available). All ages. Women.

**Figure 15:**
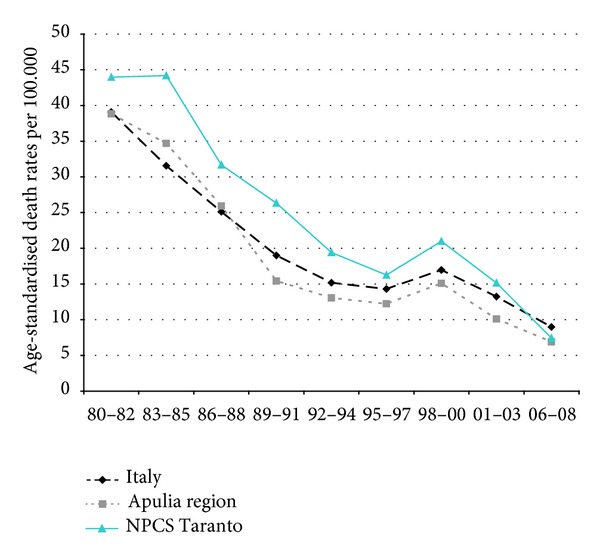
Acute respiratory diseases. Trends in age-standardised (Italian Census 2001) death rates per 100.000, from selected causes of death (1980–2008) (2004-2005 data were not available). All ages. Women.

**Figure 16:**
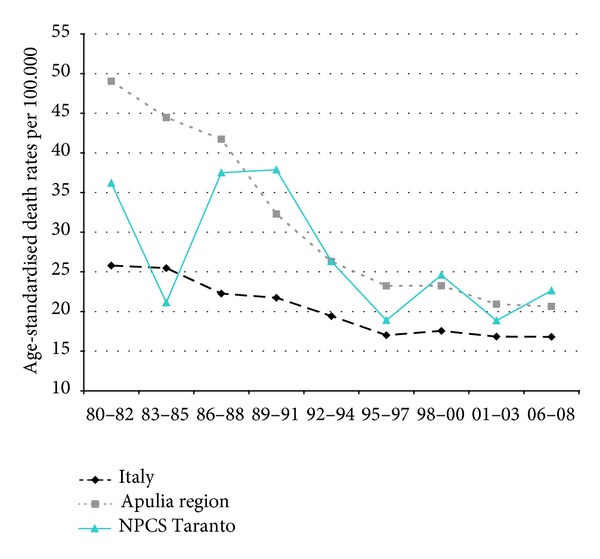
Chronic respiratory diseases. Trends in age-standardised (Italian Census 2001) death rates per 100.000, from selected causes of death (1980–2008) (2004-2005 data were not available). All ages. Women.

Finally, mortality from respiratory diseases (overall, acute, and chronic) showed a decline in Italy, Apulia, and Taranto site, but the rates in Taranto are higher than in Italy (Figures 14, 15, and 16).

The results indicate that some differential between Taranto and other areas are emerging in recent years; it may be attributed to the presence for many years of pollutants in the environment, given that no remediation has been performed in the Taranto area.

#### 3.2.3. Infant Mortality

**Figure 17:**
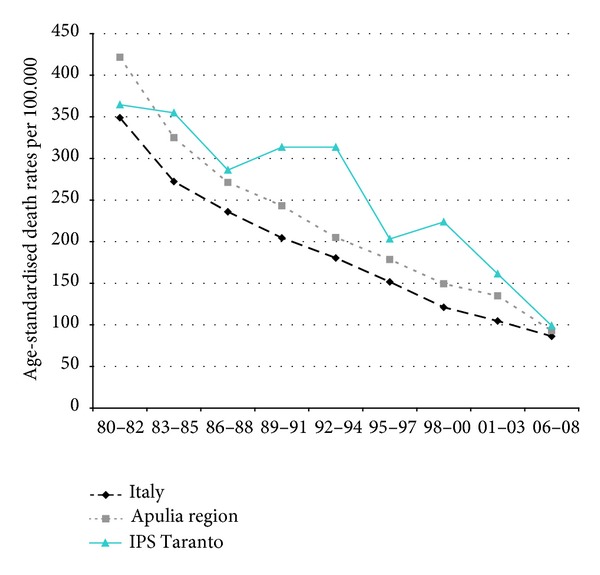
Overall mortality. Trends in age-standardised (Italian Census 2001) death rates per 100.000 (1980–2008) (2004-2005 data were not available). Infant Mortality (0 yrs).

Infant mortality showed a steady decline both in Italy and Apulia; SDRs in Taranto were decreasing, but they stayed higher in Taranto than in Apulia and Italy (Table S4 and Figure 17).

### 3.3. Cancer Incidence 2006-2007

In Taranto NPCS (Table 4) excesses were observed among both males and females when using for comparison both the rates of South and Islands macroarea and of the province (Taranto and Statte excluded) for all tumors and a number of tumor sites (stomach, colon-rectum, liver, pancreas, lung, mesothelioma, skin melanoma, kidney and other unspecified genitourinary organs, and leukemia). Among males an increase was present for prostate cancer and among females for breast cancer. For most sites, the excesses were confirmed when province rates (Taranto and Statte excluded) were used for reference, thus supporting a major health impact among residents in Taranto NPCS.

### 3.4. Mortality 1998–2008 and Morbidity 1998–2010 of the Residential Cohort

**Figure 18:**
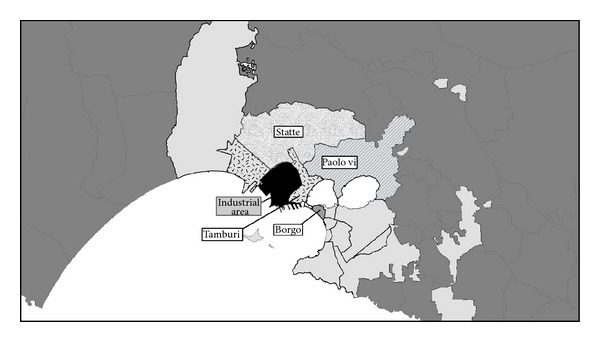
Taranto study area, districts.

The study area was divided into 9 districts in Taranto city and 2 municipalities (Massafra and Statte).  Figure 18 shows the districts investigated and the location of the industrial area. We considered that the districts located close to the industrial zone were the most areas affected by environmental pollution, especially considering the prevailing winds from northwest. The *exposed *districts were (1) Tamburi (we also included in this category the small districts of Isola, Porta Napoli, and Lido Azzurro), (2) Borgo, (3) Paolo VI, and (4) Statte. All the other districts were considered the reference zone (Italia-Montegragnano, San Vito-Lama-Carelli, Salinella, Solito, Corvisea, Talsano, Tre Carrare-Battisti, and Massafra).

A total of 321,356 people (157,031 males and 164,325 females) were enrolled in the cohort. At the time of the enrolment (January 1, 1998), 84.9% of the subjects were already resident in the study area, and 39.1% of them had been residing at the same address for more than 20 years. As far as socioeconomic status is concerned, 35% of the cohort members were in the low SEP category and 21.4% in the high SEP category. The social distribution in the different districts was heterogeneous, with elevated proportion of high social level (62.2%) in some districts in the reference area (San Vito, Lama, Carelli) and low social level in Tamburi (69.4%) and Paolo VI (64.3%). In the Tamburi and Paolo VI districts there was a higher proportion of subjects with previous employment at the steel industry than in other areas.

A total of 3,384,302 person years were accumulated for the cohort. At the end of follow-up (December 31, 2010), 76.6% of the cohort members were alive and resident in the study area, 14.6% had moved, and 28,171 individuals (8.8%) had died. The cause of death was known only for 23,004 individuals deceased by 2008. Only 2.3% of the study subjects were born abroad, and most of the cohort members were born in Taranto (81.6%) and in southern Italy (93.5%).

An analysis of the mortality differences by socioeconomic status (low *versus *high category) shows (data not in Tables) in both genders a higher mortality for all causes (Hazard ratio—HR 1.25, 95% CI 1.19–1.31 among males and HR 1.18, 95% CI 1.13–1.24 among females), cardiovascular diseases (HR 1.14, 95% CI 1.04–1.25 among males and HR 1.21, 95% CI 1.11–1.32 among females), respiratory diseases (HR 1.89, 95% CI 1.55–2.29 among males and HR 1.38, 95% CI 1.11–1.72 among females), and digestive diseases (HR 1.46, 95% CI 1.19–1.79 among males and HR 1.56, 95% CI 1.24–1.95 among females). Socioeconomic differences in mortality were observed among males for all neoplasms (HR 1.26, 95% CI 1.15–1.37) and cancer of the stomach (HR 1.69, 95% CI 1.10–2.59), larynx (HR 3.32, 95% CI 1.55–7.09), lung (HR 1.40, 95% CI 1.20–1.64), and bladder (HR 1.55, 95% CI 1.08–2.23). The results for hospitalizations confirm the increased risks among subjects in the low socioeconomic category when compared with those in the highest socioeconomic group.

Tables 5 and 6 show, respectively, for males and females, cause-specific mortality in the *exposed *subareas, that is, Tamburi, Borgo, Paolo VI, and Statte, compared with mortality observed in the reference ones. After adjusting for socioeconomic status, the *exposed *subareas (Tamburi, Borgo, Paolo VI, and Statte) have a higher mortality for all causes in comparison with the reference in males (in particular, Paolo VI and Tamburi). The most notable increases in mortality among males were in Paolo VI, with 42% excess for all malignant neoplasms (especially lung cancer, +76%), diseases of the cardiovascular (+28%), respiratory (+64%) and digestive (+47%) systems. In Tamburi, an excess was observed among males for all malignant neoplasms (+11%) and cardiovascular diseases (+10%), specifically ischemic heart diseases (+20%). Among females, in Paolo VI, excesses are present for all cancers (+23%), in particular lung, pleural and liver cancer, cardiovascular diseases (+18%), chronic obstructive pulmonary disease (COPD), and digestive system diseases. In Tamburi, excesses were present among females for cardiovascular diseases (+15%), in particular ischemic heart diseases, COPD (+39%), and renal diseases (+57%).

Tables 7 and 8 display the results for hospital admissions. The hospitalization analysis confirms the mortality results, documenting the major health impact on residents in Tamburi and Paolo VI areas, where excesses were observed among males for a number of causes such as lung cancer (29% and 61% in Tamburi and Paolo VI, resp.), neurological (26% and 43% in Tamburi and Paolo VI, resp.), cardiovascular (18% and 32% in Tamburi and Paolo VI, resp.), respiratory (36% and 52% in Tamburi and Paolo VI, resp.), and renal diseases (35% both in Tamburi and Paolo VI). Among females, similar excesses were observed for cardiovascular diseases (15 and 31% in Tamburi and Paolo VI, resp.), respiratory diseases (28% and 39% in Tamburi and Paolo VI, resp.), digestive diseases (18% and 25% in Tamburi and Paolo VI, resp.), and renal diseases (47% and 35% among males in Tamburi and Paolo VI, resp.). In Paolo VI, pleural (235%) and breast cancer (33%) were also in excess among females.

## 4. Discussion

The health impact of residence in Taranto NPCS was investigated using different epidemiological approaches: geographical (mortality and cancer incidence at municipality level), historical (mortality time trends at municipality level), and residential cohort studies (mortality and hospital discharge records at individual level). We adopted an analysis at small-area scale which includes a mix of small area and individual based data. The design issues relevant to this type of investigation have been thoroughly examined by Elliott and Savitz [27], some of them are briefly examined in the following paragraphs.

In environmental epidemiology, exposure ascertainment is a key phase because the exposure/s affecting the study population should ideally be described in detail, while in most instances the available exposure information is indirect and qualitative. In ecological investigations, the exposure/s can be a single event from a point emission source of some contaminants; more often the contaminants are heterogeneous mixture progressively polluting different matrices in the area. For example, in SENTIERI Project the sources of *environmental exposures* were abstracted from the legislative decrees defining sites' boundaries and fixed on the basis of the possible sources of contamination (e.g., chemical industry, steel plants, and landfills). A further limitation lies in the implicit assumption that all residents in the area under investigation experience the same exposures, while exposure variability is likely to be substantial, due to many factors (e.g., concentration of contaminants and their diffusion to soil and water, distance of residence from polluting sources). The possible consequences of such nondifferential exposure misclassification are complex, and the direction of the resulting bias is not predictable [28]. In addition, information exposure source/s with possible health impact, such as concurrent air pollution from road traffic and exposures in the occupational setting, are often not available. Finally, vital statistics are accessible for a given administrative area whose boundaries hardly correspond to the distribution of environmental pollutants, so that the misclassification of exposure (and loss of statistical power) is common. A more detailed description of these limitations of ecological study design is available [15, 28, 29].

Exposure ascertainment is a critical issue in ecological investigations as well as in studies based on individuals, as cohort of residents are. In this case residential history, overtime information on exposure/s in different residences and different environmental matrices, daily variability, and seasonal variability should be available to classify subjects in different exposure categories. Obtaining individual-based measures of exposure as described above is clearly infeasible, and modeling of exposures, ranging from simple measures such as distance from a point source or distance to nearest road to more complex estimation, for example, dispersion modeling around a point source, is used. The possible exposure misclassification in such approaches has been discussed in [27].

As far as outcome measures are concerned, many studies of environmental healthin polluted areas consider mortality, based on death records. However, the analysis of hospital discharge records, *ad hoc* registry data of specific pathologies (e.g., cancer, congenital malformations) can give a better picture of the health profile of residents in NPCSs. The key issue is that, whatever the outcome under investigation, databases need to be validated for use in epidemiological studies.

Reporting the event of death is usually exhaustive; therefore the overall mortality can be analyzed with confidence [30]. In Italy, validity of cause of death certification has been documented for specific diseases [31–34]. The validity of hospital discharge records (HDRs), indicators of hospital activity, has not been systematically evaluated, although in Italy some critical aspects of this novel utilization of HDRs have been examined [35].

Another crucial aspect in environmental health studies is that factors such as socioeconomic status, occupational exposures/s, and individual lifestyles can have an etiologic role on the health effects under study thus possibly confounding the exposure-disease relationships.

For a review of adjustment for socioeconomic status using census data in ecological studies of environment and health refer to Pasetto et al, 2010 [36]. To account for deprivation in SENTIERI Project, mortality data both crude and adjusted were analyzed using an *ad hoc* built deprivation index [25]. Also in individual-based studies, the analyses can be adjusted for socioeconomic factors, usually with an aggregate indicator based on residence address. In the present cohort of residents in Taranto NPCS SEP was assigned to each participant on the basis of the geocoded addresses at the beginning of the follow-up.

Occupational exposure/s are also potential confounders in environmental health studies. The ecological components of the present investigation are affected by this limitation, while for the cohort study individual occupational history was traced through the national insurance company (INPS) database, and the subcohort of individuals employed in industries located in the area was identified indicating a high proportion of past employment at the steel plant among residents in Tamburi and Paolo VI.

Again on this topic, with specific reference to pleural mesothelioma, it should be noted that the Italian National Mesothelioma Registry analysis of residential asbestos exposure showed that steel mills and iron foundries were the second most frequent sources of asbestos in the neighborhood (after asbestos-cement factories), ranking equal to asbestos textiles production [37]. Furthermore a case-control study in the area of Taranto (HDR, 1998–2002) adjusted for occupational exposures [11], showed an increase in malignant pleural neoplasms among residents close to the steel mill (or 1.62, 95% CI 0.37–7.10, 11 cases) and the coke plant (or 2.18, 95% CI 0.31–15.31, 9 cases).

Another aspect to consider in environmental epidemiology is that large populations are needed to study many of the health concerns of greatest interest. In ecological investigations, the reference populations should be selected balancing the need for comparability of study and reference populations for factors other than the environmental exposure/s with possible health impact (socioeconomic status and lifestyle factors as diet and tobacco use) and the requirement of sufficiently numerous populations to have stable reference rates also for rare diseases. These needs are satisfied in the present investigation where national, macroregional, and regional populations were used for comparison in the mortality, time trend, and cancer incidence analysis. In the cohort study an internal comparison was carried out, and the reference population was composed of residents in districts distant from the industrial area.

For chronic diseases including most cancers, latency effects are important, such that exposures experienced many years previously, or accumulated exposures, may be crucial. In Taranto NPCS, the mortality (1995–2009) and the time trends analyses (1980–2008) show consistent results and cover a time span which should encompass latency effects. Analogously in the cohort of residents, most subjects were present at enrolment in 1998 (85%), and half of them had a residence duration of 10 or more years.

A brief comment on the use of 90% CI in our analyses is needed. In this respect we refer to Sterne and Smith [38], who affirm that confidence intervals for the main results should always be included, but 90% rather than 95% levels should be used. CI should not be used as a surrogate means of examining significance at the conventional 5% level. Interpretation of CI should focus on the implications (clinical importance) of the range of values in the interval.

## 5. Conclusions

In Taranto NPCS, mortality data at municipality level analyzed, in the context of SENTIERI Project, time trend analysis and cancer incidence results coherently showed, in both genders, excess risks for a number of causes of death, among them: all causes, all cancers, lung cancer, cardiovascular, and respiratory diseases, both acute and chronic. For these causes, an etiologic role of environmental exposure present in Taranto NPCS can be supported on the basis of *a priori* evaluation of the epidemiological evidence completed in SENTIERI. In the cohort study among residents in the districts nearer to the industrial area, excess mortality/morbidity risks were shown for natural cause, cancers, cardiovascular, and respiratory diseases. These excesses were also observed in low socioeconomic position groups compared to high ones, some of them could be explained on the basis of previous employment of residents in industries active in the study area.

As discussed above the present results for Taranto NPCS are based on sound study design and valid data, which make a low potential for bias and help to strengthen etiologic inference. The present findings further corroborate the need to promptly proceed with environmental cleanup interventions.

It should not be disregarded the fact that most diseases showing an increased risk have multifactorial etiology, therefore interventions of proven efficacy, such as smoking cessation, food education, measures for cardiovascular risk reduction, and breast cancer screening programs, should be planned. To build a climate of confidence and trust between citizens and public institutions, study results and public health actions are to be communicated objectively and transparently.

## Supplementary Material

Table S1: The International Classification of Disease (ICD) is revised approximately every 10 years. In Italy deaths have been coded according ICD-9 until 2002, since 2003, ICD-10 has been adopted. Because ICD-10 differs from ICD-9 in several respects comparability studies (also called bridge-coding) measure the effects of a new revision of the ICD on the comparability with the previous revision of mortality statistics by cause of death. The key element of a comparability study is the “comparability ratio”, which is derived from the dual classification. Table S1 displays the comparability results for some causes investigated in the time trend analysis.
Tables S2-S4 display number of deaths and standardized (Italian Census 2001) rates per 100.000 for selected causes of death in NPCS of Taranto, in Apulia Region and in Italy. 1980-2008 (2004-2005 data not available); all ages; men, women, infants . Time trends for these date are displayed in Figures 1-17.

## Figures and Tables

**Table 1:** SENTIERI—Taranto NPCS. Mortality for the main causes of death. Number of observed cases (Obs), standardized mortality ratio crude (SMR), and adjusted for deprivation (SMR ID); 90% IC: 90% confidence interval; regional references: 1995–2002 and 2003–2009. Males and females.

Causes of death (ICD IX)	1995–2002						2003–2009*					
	Males			Females			Males			Females		
	OBS	SMR (90% CI)	SMR ID (90% CI)	OBS	SMR (90% CI)	SMR ID (90% CI)	OBS	SMR (90% CI)	SMR ID (90% CI)	OBS	SMR (90% CI)	SMR ID (90% CI)
All causes (1–999.9)	7585	109 (107–111)	107 (105–109)	7104	107 (105–109)	107 (105–109)	4936	114 (111–117)	111 (108–113)	4847	108 (105–110)	107 (104–109)
All neoplasms (140.0–239.9)	2529	115 (112–119)	113 (109–116)	1716	113 (108–117)	112 (108–117)	1650	114 (110–119)	111 (106–115)	1208	113 (108–118)	111 (106–116)
Diseases of the circulatory system (401.0–405.9)	2654	105 (102–108)	103 (99–106)	3118	101 (98–104)	100 (97–103)	1645	114 (109–119)	109 (105–114)	1968	104 (100–108)	103 (99–107)
Diseases of the respiratory system (460.0–519.9)	666	107 (100–114)	107 (100–114)	406	113 (104–123)	111 (102–120)	447	117 (108–126)	112 (103–121)	268	104 (94–115)	105 (95–116)
Diseases of the digestive system (520.0–579.9)	442	114 (105–123)	114 (106–124)	472	142 (132–153)	141 (131–153)	283	147 (133–162)	136 (123–150)	233	119 (106–132)	117 (104–130)
Diseases of the genitourinary system (580.0–629.9)	101	92 (78–109)	97 (82–115)	107	89 (75–104)	91 (77–108)	71	94 (77–115)	101 (82–123)	85	89 (74–107)	87 (72–104)

*2004-2005 not available from ISTAT.

**Table 2:** SENTIERI—Taranto NPCS. Mortality for causes of death with limited evidence of association with *environmental exposures* in Taranto NPCS. Number of observed cases (Obs), standardized mortality ratio crude (SMR), and adjusted for deprivation (SMR ID); 90% IC: 90% confidence interval; regional references: 1995–2002 and 2003–2009. Males and females.

Causes of death (ICD IX)	1995–2002						2003–2009*					
	Males			Females			Males			Females		
	OBS	SMR (90% CI)	SMR ID (90% CI)	OBS	SMR (90% CI)	SMR ID (90% CI)	OBS	SMR (90% CI)	SMR ID (90% CI)	OBS	SMR (90% CI)	SMR ID (90% CI)
Malignant neoplasm of trachea bronchus and lung (162.0–162.9)	840	130 (122–137)	119 (112–126)	121	135 (115–157)	130 (111–151)	516	133 (124–143)	123 (114–132)	97	130 (109–153)	121 (101–143)
Malignant pleural neoplasm (163.0–163.9)	83	521 (430–625)	293 (242–352)	14	242 (147–379)	190 (115–297)	44	519 (397–667)	272 (208–350)	12	311 (180–505)	210 (121–340)
Diseases of the respiratory system (460.0–519.9)	666	107 (100–114)	107 (100–114)	406	113 (104–123)	111 (102–120)	447	117 (108–126)	112 (103–121)	268	104 (94–115)	105 (95–116)
Acute diseases of the respiratory system (460.0–466.9, 480.0–487.9)	125	156 (134–181)	149 (127–173)	135	145 (125–167)	138 (119–159)	50	136 (106–172)	137 (107–174)	58	112 (89–140)	115 (91–143)
Chronic diseases of the respiratory system (491.0–492.9, 494.0–496.9)	388	96 (88–105)	97 (89–105)	151	92 (80–105)	92 (80–105)	322	116 (106–127)	110 (100–121)	149	104 (90–119)	100 (87–114)
Asthma (493.0–493.9)	9	41 (22–72)	42 (22–73)	11	73 (41–121)	68 (38–113)	0			1	25 (1–118)	29 (1–137)

*2004-2005 not available from ISTAT.

**Table 3:** SENTIERI—Taranto NPCS. Mortality for causes of death with limited evidence of association with *environmental exposures* in Taranto NPCS. Number of observed cases (Obs), standardized mortality ratio crude (SMR), and adjusted for deprivation (SMR ID); 90% IC: 90% confidence interval; regional references: 1995–2002 and 2003–2009. Males and females combined.

Causes of death (age classes) (ICD IX)	1995–2002			2003–2009*		
	Total			Total		
	OBS	SMR (90% CI)	SMR ID (90% CI)	OBS	SMR (90% CI)	SMR ID (90% CI)
Congenital anomalies (all ages) (740.0–759.9)	59	115 (91–142)	117 (93–145)	20	82 (54–119)	93 (62–135)
Certain conditions originating in the perinatal period (0-1) (760.0–779.9)	79	135 (111–162)	121 (100–146)	37	165 (123–218)	147 (110–193)
Acute diseases of the respiratory system (0–14) (460.0–466.9, 480.0–487.9)	4	96 (33–219)	95 (33–219)	<3	—	—
Asthma (0–14) (493.0–493.9)	<3	—	—	<3	—	—

*2004-2005 not available from ISTAT.

**Table 4:** Cancer incidence Taranto NPCS. Number of observed cases (Obs), standardized incidence ratio crude (SIR); 90% IC: 90% confidence interval; Reference SIR: macroarea South and Islands 2005–2007; Reference SIR: Taranto province excluding NPCS municipalities (TA-NPCS) 2006-2007.

Cancer site	Males			Females		
	OBS	SIR-macroarea South and Islands (90% CI)	SIR (TA-NPCS) (90% CI)	OBS	SIR-macro-area South and Islands (90% CI)	SIR (TA-NPCS) (90% CI)
Head and neck	52	98 (77–123)	131 (103–165)	13	96 (57–153)	134 (79–213)
Stomach	51	130 (102–164)	117 (91–148)	43	167 (127–215)	224 (171–289)
Colon and rectum	145	112 (97–129)	122 (106–140)	131	115 (99–133)	121 (104–140)
Liver	64	110 (88–135)	140 (113–172)	36	110 (82–145)	175 (130–231)
Pancreas	28	100 (71–137)	135 (96–185)	31	113 (82–153)	129 (93–174)
Lung	245	144 (129–160)	150 (135–167)	47	117 (90–149)	148 (114–189)
Skin melanoma	35	214 (158–284)	193 (143–256)	23	143 (98–203)	120 (82–170)
Mesothelioma	21	429 (287–618)	256 (172–369)	3	197 (53–509)	81 (22–209)
Breast	—	—	—	317	130 (118–143)	124 (113–136)
Prostate	204	129 (112–148)	121 (105–139)	—	—	—
Testis	12	109 (63–177)	79 (46–128)	—	—	—
Uterus, cervix	—	—	—	14	93 (56–145)	88 (53–138)
Uterus, body	—	—	—	69	134 (109–164)	188 (152–230)
Ovary	—	—	—	35	119 (88–158)	81 (60–107)
Kidney and other unspecified urinary organs	51	164 (128–207)	201 (157–254)	18	119 (77–176)	114 (74–169)
Bladder	188	141 (125–159)	136 (120–153)	23	62 (42–88)	92 (63–130)
Brain and CNS (malignant)	16	86 (54–131)	88 (55–134)	12	78 (45–126)	65 (37–105)
Thyroid	23	169 (115–239)	126 (86–179)	71	152 (124–185)	94 (76–115)
Hodgkin lymphoma	6	88 (38–174)	63 (27–124)	8	131 (65–236)	70 (35–126)
Non-Hodgkin lymphoma	42	119 (90–154)	160 (122–207)	28	88 (63–121)	143 (102–196)
Myeloma	18	140 (90–208)	135 (87–200)	15	107 (66–165)	97 (60–149)
Leukemia	30	108 (78–146)	82 (59–111)	37	164 (122–216)	103 (77–136)
All tumors excluding skin, nonmalignant brain, and CNS	1338	131 (125–137)	130 (124–136)	1084	126 (120–132)	121 (115–127)

**Table 5:** Association between district and cause-specific mortality (HR, 90% CI) (males, 1998–2008). Hazard Ratio (HR) from the Cox model stratified by calendar period and adjusted for age (underlying time) and socioeconomic position.

Cause of Death (ICD-9-CM)	Reference districts	Tamburi				Borgo				Paolo VI				Statte			
	*n* = 107,909	*n* = 14,067				*n* = 16,312				*n* = 10,097				*n* = 8,283			
	*n*	*n*	HR	90% CI		*n*	HR	90% CI		*n*	HR	90% CI		*n*	HR	90% CI	
All causes (001–999)*	9.378	1.470	1,12	1,07	1,18	1.973	1,07	1,03	1,12	684	1,27	1,19	1,36	654	1,08	1,01	1,15
Malignant cancers (140–208)	2.650	400	1,11	1,01	1,21	505	1,00	0,93	1,09	223	1,42	1,26	1,59	178	1,05	0,93	1,20
Stomach (151)	126	22	1,24	0,83	1,84	28	1,20	0,85	1,71	12	1,62	0,97	2,69	7	0,85	0,45	1,61
Colorectal (153-154)	220	19	0,62	0,41	0,92	48	1,13	0,87	1,48	12	1,07	0,65	1,75	11	0,79	0,48	1,32
Trachea, bronchus, and lung (162)	829	127	1,09	0,93	1,28	150	0,97	0,84	1,13	94	1,76	1,46	2,11	61	1,12	0,90	1,39
Pleura (163)	80	12	1,09	0,64	1,85	16	1,08	0,68	1,71	6	1,19	0,59	2,42	2	0,39	0,12	1,27
Prostate (185)	244	45	1,42	1,07	1,89	53	0,98	0,76	1,26	8	0,84	0,46	1,52	12	0,85	0,52	1,39
Bladder (188)	189	34	1,20	0,87	1,66	29	0,73	0,53	1,02	13	1,45	0,90	2,35	13	1,17	0,73	1,87
Kidney (189)	21	5	2,23	0,93	5,33	5	1,30	0,56	2,98	2	1,85	0,53	6,39	5	3,69	1,62	8,45
Brain and other parts of CNS (191-192; 225)	83	15	1,37	0,85	2,23	18	1,23	0,80	1,90	10	1,64	0,93	2,88	6	1,07	0,53	2,14
Lymphatic and hematopoietic tissue (200–208)	212	27	1,05	0,74	1,49	41	1,07	0,81	1,43	14	1,01	0,64	1,60	9	0,69	0,39	1,20
Neurological diseases (330–349)	190	28	1,09	0,77	1,54	30	0,76	0,54	1,05	7	0,72	0,38	1,36	15	1,32	0,85	2,06
Cardiovascular diseases (390–459)	2.442	378	1,10	1,00	1,21	551	1,02	0,94	1,10	147	1,28	1,11	1,47	137	0,93	0,81	1,08
Cardiac diseases (390–429)	1.688	260	1,09	0,98	1,23	387	1,03	0,94	1,13	106	1,27	1,08	1,51	84	0,82	0,68	0,99
Ischemic heart diseases (410–414)	733	116	1,20	1,01	1,42	152	1,04	0,89	1,20	56	1,37	1,09	1,73	30	0,66	0,49	0,90
Cerebrovascular diseases (430–438)	551	86	1,06	0,87	1,29	109	0,87	0,73	1,04	25	1,07	0,76	1,50	43	1,34	1,04	1,75
Respiratory diseases (460–519)	697	122	1,08	0,91	1,27	177	1,05	0,91	1,20	48	1,64	1,28	2,11	59	1,46	1,17	1,82
COPD (490–492, 494, 496)	469	93	1,17	0,96	1,41	110	0,94	0,79	1,12	32	1,70	1,25	2,32	39	1,44	1,10	1,90
Diseases of the digestive system (520–579)	527	81	1,06	0,86	1,30	111	1,07	0,90	1,27	47	1,47	1,14	1,90	27	0,79	0,57	1,09
Renal diseases (580–599)	146	27	1,36	0,94	1,95	37	1,13	0,83	1,54	3	0,50	0,19	1,30	9	1,00	0,57	1,76

*Referring to the period 1998–2010.

**Table 6:** Association between district and cause-specific mortality (HR, 90% CI) (females, 1998–2008). Hazard Ratio (HR) from the Cox model stratified by calendar period and adjusted for age (underlying time) and socioeconomic position.

Cause of death (ICD-9-CM)	Reference districts	Tamburi				Borgo				Paolo VI				Statte			
	*n* = 112,897	*n* = 14,625				*n* = 18,528				*n* = 9,714				*n* = 8,271			
	*n*	*n*	HR	90% CI		*n*	HR	90% CI		*n*	HR	90% CI		*n*	HR	90% CI	
All causes (001–999)*	9.015	1.479	1,09	1,04	1,15	2.482	1,01	0,97	1,05	489	1,28	1,18	1,38	547	1,06	0,98	1,14
Malignant cancers (140–208)	1.900	230	0,84	0,75	0,95	434	0,95	0,87	1,04	126	1,23	1,06	1,44	102	0,92	0,78	1,08
Stomach (151)	96	20	1,52	0,99	2,34	24	1,01	0,69	1,48	7	1,47	0,76	2,83	7	1,31	0,69	2,51
Colorectal (153-154)	226	23	0,62	0,43	0,90	45	0,78	0,59	1,02	16	1,35	0,87	2,08	7	0,54	0,29	1,02
Trachea, bronchus and lung (162)	144	15	0,76	0,48	1,20	34	1,06	0,77	1,46	13	1,71	1,05	2,79	6	0,68	0,34	1,34
Pleura (163)	20	2	0,66	0,19	2,31	6	1,16	0,53	2,52	3	2,95	1,03	8,46	0			
Breast (174)	349	41	0,92	0,69	1,22	89	1,18	0,96	1,44	28	1,29	0,93	1,79	22	1,04	0,72	1,49
Bladder (188)	33	7	1,23	0,61	2,50	12	1,13	0,64	1,97	2	1,29	0,39	4,34	1	0,58	0,11	3,06
Kidney (189)	17	0				3	0,87	0,30	2,49	0	0,00	0,00	0,00	1	1,06	0,19	5,79
Brain and other parts of CNS (191-192; 225)	90	6	0,48	0,24	0,97	17	0,85	0,55	1,33	4	0,67	0,29	1,57	7	1,30	0,68	2,48
Lymphatic and hematopoietic tissue (200–208)	202	22	0,74	0,50	1,08	33	0,65	0,47	0,88	11	0,98	0,59	1,65	11	0,99	0,60	1,66
Neurological diseases (330–349)	216	35	1,08	0,79	1,47	50	0,83	0,64	1,08	13	1,68	1,04	2,71	11	0,87	0,52	1,45
Cardiovascular diseases (390–459)	2.945	529	1,15	1,06	1,24	876	0,93	0,88	1,00	125	1,18	1,01	1,37	166	0,98	0,86	1,12
Cardiac diseases (390–429)	1.910	371	1,24	1,12	1,37	623	1,04	0,96	1,12	84	1,22	1,01	1,47	90	0,81	0,68	0,97
Ischemic heart diseases (410–414)	565	124	1,46	1,23	1,73	171	1,02	0,88	1,18	24	1,15	0,81	1,63	27	0,86	0,62	1,19
Cerebrovascular diseases (430–438)	820	122	0,93	0,79	1,10	207	0,77	0,68	0,88	35	1,19	0,90	1,59	62	1,38	1,11	1,71
Respiratory diseases (460–519)	476	82	1,09	0,89	1,34	169	1,09	0,94	1,26	22	1,26	0,88	1,82	34	1,28	0,95	1,71
COPD (490–492, 494, 496)	220	49	1,39	1,06	1,83	70	0,97	0,77	1,21	16	2,14	1,38	3,29	14	1,16	0,74	1,83
Diseases of the digestive system (520–579)	484	77	0,95	0,77	1,16	119	0,88	0,74	1,04	29	1,43	1,04	1,97	30	1,13	0,83	1,54
Renal diseases (580–599)	166	38	1,57	1,15	2,14	49	1,01	0,77	1,33	10	1,68	0,98	2,90	11	1,12	0,67	1,87

*Referring to the period 1998–2010.

**Table 7:** Association between district and cause-specific hospitalization (HR, 90% CI) (males, 1998–2010). Hazard Ratio (HR) from the Cox model stratified by calendar period and adjusted for age (underlying time) and socioeconomic position.

Diagnosis (ICD-9-CM)	Reference districts	Tamburi				Borgo				Paolo VI				Statte			
	*n* = 108,272	*n* = 14,067				*n* = 16,312				*n* = 10,097				*n* = 8,283			
	*n*	*n*	HR	90% CI		*n*	HR	90% CI		*n*	HR	90% CI		*n*	HR	90% CI	
Malignant cancers (140–208)	4.818	685	1,12	1,05	1,21	861	1,06	0,99	1,12	444	1,31	1,21	1,43	354	1,06	0,97	1,16
Stomach (151)	166	27	1,21	0,85	1,73	30	1,05	0,75	1,46	19	1,63	1,09	2,46	15	1,29	0,83	2,02
Colorectal (153-154)	520	52	0,86	0,67	1,10	73	0,87	0,70	1,07	28	0,82	0,59	1,13	39	1,08	0,82	1,41
Trachea, bronchus, and lung (162)	866	149	1,29	1,10	1,50	156	1,06	0,92	1,22	101	1,61	1,34	1,92	60	0,98	0,78	1,22
Pleura (163)	75	18	1,80	1,14	2,84	18	1,38	0,89	2,15	7	1,44	0,74	2,79	4	0,77	0,33	1,79
Connective and other soft tissue (171)	32	4	0,91	0,37	2,24	10	1,80	0,98	3,31	5	1,66	0,74	3,72	3	1,30	0,48	3,52
Prostate (185)	639	80	1,10	0,90	1,35	113	1,04	0,88	1,24	40	0,98	0,75	1,29	51	1,22	0,96	1,56
Testis (186)	49	3	0,42	0,15	1,13	9	1,23	0,67	2,25	2	0,40	0,12	1,34	5	1,31	0,60	2,84
Bladder (188)	787	106	1,12	0,94	1,34	121	0,90	0,77	1,06	84	1,62	1,34	1,97	50	0,95	0,74	1,20
Kidney (189)	149	23	1,26	0,85	1,85	37	1,47	1,08	1,99	15	1,41	0,89	2,22	14	1,37	0,86	2,17
Brain and other parts of CNS (191-192; 225)	187	23	0,98	0,67	1,43	30	1,01	0,73	1,41	14	0,89	0,56	1,41	16	1,19	0,77	1,83
Lymphatic and hematopoietic tissue (200–208)	400	58	1,20	0,94	1,53	80	1,26	1,03	1,55	35	1,13	0,84	1,52	24	0,84	0,60	1,19
Neurological diseases (330–349)	1.850	329	1,26	1,14	1,40	337	1,11	1,00	1,22	226	1,43	1,27	1,61	140	1,04	0,90	1,20
Cardiovascular diseases (390–459)	14.504	2.078	1,18	1,13	1,23	2.457	1,03	1,00	1,07	1.388	1,32	1,26	1,39	1.076	1,06	1,01	1,12
Cardiac diseases (390–429)	9.866	1.411	1,20	1,15	1,27	1.699	1,04	0,99	1,09	957	1,41	1,33	1,49	752	1,10	1,04	1,17
Acute coronary events (410-411)	2.328	310	1,13	1,02	1,26	396	1,09	0,99	1,19	241	1,39	1,24	1,56	167	1,00	0,87	1,14
Heart failure (428)	1.878	317	1,21	1,09	1,34	375	1,03	0,94	1,14	180	1,54	1,35	1,76	119	0,97	0,83	1,13
Cerebrovascular diseases (430–438)	3.124	525	1,30	1,19	1,41	581	1,04	0,96	1,12	211	1,01	0,89	1,13	226	1,09	0,97	1,22
Respiratory diseases (460–519)	8.906	1.836	1,36	1,30	1,43	1.493	1,01	0,96	1,06	1.255	1,52	1,44	1,60	752	1,12	1,05	1,19
Acute respiratory infections (460–466, 480–487)	3.769	961	1,51	1,42	1,61	746	1,19	1,11	1,27	625	1,54	1,44	1,66	315	1,05	0,95	1,15
COPD (490–492, 494, 496)	2.350	450	1,31	1,20	1,43	379	0,85	0,78	0,93	252	1,60	1,43	1,79	203	1,29	1,14	1,46
Diseases of the digestive system (520–579)	15.628	2.465	1,20	1,15	1,24	2.301	0,94	0,91	0,98	1.682	1,23	1,18	1,29	1.229	1,06	1,01	1,12
Renal diseases (580–599)	3.252	562	1,35	1,25	1,46	521	0,98	0,90	1,06	331	1,35	1,22	1,49	282	1,22	1,10	1,36

**Table 8:** Association between district and cause-specific hospitalization (HR, 90% CI) (females, 1998–2010). Hazard Ratio (HR) from the Cox model stratified by calendar period and adjusted for age (underlying time) and socioeconomic position.

Diagnosis (ICD-9-CM)	Reference districts	Tamburi				Borgo				Paolo VI				Statte			
	*n* = 113,187	*n* = 14,625				*n* = 18,528				*n* = 9,714				*n* = 8,271			
	*n*	*n*	HR	90% CI		*n*	HR	90% CI		*n*	HR	90% CI		*n*	HR	90% CI	
Malignant cancers (140–208)	3.878	530	1,03	0,95	1,12	713	0,91	0,85	0,97	295	1,17	1,06	1,29	225	0,91	0,81	1,02
Stomach (151)	129	23	1,26	0,85	1,85	24	0,89	0,61	1,28	7	1,06	0,55	2,02	9	1,10	0,62	1,94
Colorectal (153-154)	483	60	0,93	0,74	1,18	62	0,62	0,50	0,78	35	1,21	0,90	1,62	17	0,58	0,39	0,88
Trachea, bronchus, and lung (162)	149	17	0,89	0,57	1,37	30	0,94	0,67	1,33	10	1,04	0,60	1,80	5	0,53	0,25	1,12
Pleura (163)	23	3	0,87	0,31	2,44	2	0,44	0,13	1,48	4	3,35	1,31	8,54	2	1,25	0,37	4,22
Breast (174)	990	127	1,03	0,87	1,21	179	0,98	0,86	1,12	94	1,33	1,11	1,59	59	0,89	0,72	1,11
Vescica (188)	146	22	1,12	0,76	1,66	37	1,16	0,85	1,58	5	0,61	0,29	1,29	5	0,56	0,27	1,19
Kidney (189)	78	10	0,95	0,53	1,68	13	0,84	0,51	1,39	5	1,06	0,49	2,28	10	2,10	1,21	3,66
Brain and other parts of CNS (191-192; 225)	204	25	0,94	0,65	1,35	30	0,77	0,55	1,06	17	1,18	0,77	1,80	17	1,27	0,84	1,93
Lymphatic and hematopoietic tissue (200–208)	398	55	1,03	0,80	1,31	58	0,72	0,57	0,91	26	1,00	0,71	1,40	24	0,95	0,67	1,35
Neurological diseases (330–349)	2.151	351	1,11	1,01	1,23	378	0,89	0,81	0,98	168	1,06	0,93	1,21	141	0,98	0,85	1,14
Cardiovascular diseases (390–459)	13.500	2.072	1,15	1,11	1,20	2.611	0,90	0,87	0,93	1.059	1,31	1,25	1,39	888	1,05	0,99	1,11
Cardiac diseases (390–429)	9.366	1.478	1,17	1,12	1,23	1.897	0,93	0,89	0,97	752	1,40	1,31	1,49	632	1,09	1,02	1,17
Acute coronary events (410-411)	1.060	197	1,32	1,16	1,51	254	1,08	0,96	1,22	88	1,42	1,18	1,72	64	1,02	0,82	1,26
Heart failure (428)	2.449	368	0,95	0,87	1,05	628	1,02	0,94	1,09	154	1,32	1,15	1,52	106	0,77	0,65	0,90
Cerebrovascular diseases (430–438)	3.595	600	1,15	1,06	1,24	728	0,84	0,79	0,90	220	1,21	1,07	1,35	212	1,01	0,90	1,13
Respiratory diseases (460–519)	6.673	1.336	1,28	1,21	1,34	1.273	1,00	0,95	1,05	813	1,39	1,31	1,49	514	1,07	0,99	1,16
Acute respiratory infections (460–466, 480–487)	3.020	705	1,39	1,29	1,49	599	1,08	1,00	1,17	422	1,37	1,25	1,49	228	0,98	0,88	1,10
COPD (490–492, 494, 496)	1.433	262	1,19	1,06	1,33	325	0,94	0,85	1,04	126	1,62	1,39	1,89	91	1,09	0,91	1,30
Diseases of the digestive system (520–579)	12.952	2.067	1,18	1,13	1,22	2.038	0,89	0,85	0,92	1.288	1,25	1,19	1,31	905	1,00	0,95	1,06
Renal diseases (580–599)	3.187	662	1,47	1,37	1,59	609	0,99	0,92	1,07	320	1,35	1,22	1,49	248	1,17	1,05	1,31

## Appendices

### A. International Classification of Diseases to Code Mortality: From ICD-9 to ICD-10

Mortality data are coded according to the International Classification of Disease (ICD) which has been revised approximately every 10 years; the purpose of the revision is to stay abreast of medical advances in terms of disease nomenclature and etiology. In Italy, deaths have been coded according to the Ninth Revision (ICD-9) until 2002 [39]; since 2003, the Tenth Revision (ICD-10) has been adopted [40].

ICD-10 differs from ICD-9 in several respects: ICD-10 is far more detailed than ICD-9, with about 12,000 categories versus about 5,000 categories; ICD-10 uses alphanumeric codes compared with numeric codes in ICD-9; some additions and modifications were made to the chapters in the ICD; and some of the coding rules and rules for selecting the underlying cause of death have been changed.

Because of these modifications, comparability studies, also called bridge-coding studies, were carried out to measure the effects of a new revision of the ICD on the comparability with the previous revision of mortality statistics by cause of death. These studies involve the dual classification of a single-year mortality data, that is, classifying the underlying cause of death on mortality records by both the new revision and the previous revision. The key element of a comparability study is the “comparability ratio”, which is derived from the dual classification.

Operationally, the comparability ratio for the cause of death *i* (*Ci*) is calculated as follows:

(A.1) $$\begin{array}{c} Ci\;=\;\frac{D_{i,\text{ICD-}10}}{D_{i,\text{ICD-}9}}, \end{array}$$

where *D*_*i*,ICD-10_ is the number of deaths due to cause *i* classified by ICD-10. *D*_*i*,ICD-9_, is the number of deaths due to cause *i* classified by ICD-9.

A comparability ratio of 1.00 indicates that the same number of deaths was assigned to cause *i* under both ICD-9 and ICD-10.

Comparability studies between ICD-9 and ICD-10 have been conducted in the USA, in Europe (by Eurostat), and also in Italy (by the Italian Census Bureau, ISTAT) [41–44]. The Italian study [44] documented that comparability ratios of the main causes of death are close to the value of one.

Table S1 presents the comparability study results that referred to some causes investigated in the time trend analysis.

In Italy the definition of municipalities has varied over time: some municipalities have been suppressed, others have been modified, and others have been created.

Generally, these modifications are not implemented at the same time by the office that registers deaths (Civil Status Office) and by the office that registers the population (General Registry office).

Therefore, it may occur that a “new” municipality (created by the subdivision of another municipality) creates first the General Registry, beginning to register its own populations, while the deaths continue to be registered by the Civil Status Office of the “old” municipality [45, 46].

In the context of SENTIERI Project the situation of all of the municipalities constituting the NPCSs has been studied [47].

As far as Taranto and Statte municipalities, that constitute Taranto NPCS, the description is as follows: until 1993, Statte was a district of Taranto; in 1994 it became a new municipality and created immediately its own General Registry Office; on the other hand, its own Civil Status office was set up some years later (1998).

To “synchronize” data regarding deaths and populations of both Statte and Taranto, we decided to attribute the population and the deaths of Statte to Taranto until 1997; since 1998, they have been attributed to Statte itself.

### B. Mortality Trends from Selected Causes in Taranto NPCS, 1980–2008

See Supplementary Tables S2, S3, and S4.
