# Supplementary material for: Environment and Health in Contaminated Sites: The Case of Taranto, Italy
Source: J Environ Public Health. 2013 Dec 24;2013:753719. doi: 10.1155/2013/753719 (PMC3886576; doi:10.1155/2013/753719)
Supplement: Supplementary file 1 — Table S1: The International Classification of Disease (ICD) is revised approximately every 10 years. In Italy deaths have been coded according ICD-9 until 2002, since 2003, ICD-10 has been adopted. Because ICD-10 differs from ICD-9 in several respects comparability studies (also called bridge-coding) measure the effects of a new revision of the ICD on the comparability with the previous revision of mortality statistics by cause of death. The key element of a comparability study is the “comparability ratio”, which is derived from the dual classification. Table S1 displays the comparability results for some causes investigated in the time trend analysis. Tables S2-S4 display number of deaths and standardized (Italian Census 2001) rates per 100.000 for selected causes of death in NPCS of Taranto, in Apulia Region and in Italy. 1980-2008 (2004-2005 data not available); all ages; men, women, infants . Time trends for these date are displayed in Figures 1-17. [file 753719.f1.docx]

Table S1: Comparability study results for a selection of causes investigated in the time trend analysis.

| **Cause** | **ICD-9 Codes** | **ICD-10 Codes** | **Comparability Rates** |
| --- | --- | --- | --- |
| Cancer (all sites) | 140-239 | C00-D48 | 1.01 |
| Cancer of trachea, bronchus and lung | 162-163 | C32-C34 | 0.98 |
| Circulatory diseases | 390-459 | I00-I99 | 0.97 |
| Ischaemic heart diseases | 410-414 | I20-I25 | 1.03 |
| Respiratory diseases | 460-519 | J00-J99 | 0.98 |

Table S2: Trends in mortality from selected causes of death in the NPCS of Taranto, in Apulia Region and in Italy. 1980-2008 (2004-2005 data not available). All ages. Men. Number of deaths and standardized (Italian Census 2001) rates per 100.000.

(a)Men-Taranto

| **All ages** | **1980-1982** | | **1983-1985** | | **1986-1988** | | **1989-1991** | | **1992-1994** | | **1995-1997** | | **1998-2000** | | **2001-2003** | | **2006-2008** | |
| --- | --- | --- | --- | --- | --- | --- | --- | --- | --- | --- | --- | --- | --- | --- | --- | --- | --- | --- |
|  | **No.** | **SDR**  **IC 90%** | **No.** | **SDR**  **IC 90%** | **No.** | **SDR**  **IC 90%** | **No.** | **SDR**  **IC 90%** | **No.** | **SDR**  **IC 90%** | **No.** | **SDR**  **IC 90%** | **No.** | **SDR**  **IC 90%** | **No.** | **SDR**  **IC 90%** | **No.** | **SDR**  **IC 90%** |
| Overall mortality | 2658 | 1873.0  (1806.9-1943.4) | 2837 | 1947.2  (1880.2-2018.7) | 2785 | 1804.6  (1724.9-1915.9) | 2809 | 1623.4  (1568.6-1682.4) | 2908 | 1566.5  (1516.6-1620.6) | 2829 | 1431.6  (1386.0-1481.3) | 2899 | 1424.0  (1381.2-1468.8) | 2813 | 1273.0  (1234.5-1313.0) | 2946 | 1258.3  (1221.6-1296.2) |
| All cancers | 690 | 387.4  (360.6-418.7) | 769 | 450.8  (419.7-486.8) | 846 | 462.4  (433.3-532.4) | 821 | 424.8  (398.0-455.9) | 870 | 419.9  (395.3-448.9) | 913 | 414.9  (391.4-442.8) | 949 | 427.1  (403.8-452.3) | 983 | 414.0  (392.2-437.3) | 992 | 397.0  (376.3-418.9) |
| *Lung cancer* | 273 | 149.6  (134.0-169.9) | 291 | 159.2  (142.2-181.3) | 288 | 146.6  (131.4-210.2) | 328 | 160.1  (145.0-179.9) | 323 | 151.8  (137.5-170.9) | 312 | 135.8  (123.0-153.3) | 322 | 139.9  (127.0-154.6) | 295 | 119.8  (125.9-155.4) | 241 | 123.8  (112.3-136.5) |
| Circulatory diseases | 1036 | 868.0  (818.1-922.4) | 1060 | 861.9  (811.8-917.0) | 890 | 674.4  (654.0-739.0) | 937 | 629.4  (591.7-671.6) | 1028 | 623.3  (589.0-661.9) | 997 | 552.4  (522.1-587.1) | 1019 | 541.4  (512.9-572.0) | 963 | 461.8  (437.4-487.9) | 965 | 431.5  (408.9-455.3) |
| *Ischaemic heart diseases* | 367 | 253.0  (227.4-283.4) | 366 | 248.8  (223.0-279.6) | 340 | 220.7  (198.2-287.5) | 389 | 221.7  (201.1-247.0) | 453 | 246.8  (226.3-271.8) | 382 | 195.8  (178.2-217.9) | 391 | 191.8  (175.2-210.4) | 406 | 184.7  (169.5-201.5) | 373 | 161.5  (147.7-176.5) |
| Respiratory diseases | 259 | 238.9  (210.6-272.2) | 249 | 229.3  (201.0-263.0) | 314 | 247.8  (221.0-317.1) | 255 | 179.3  (158.5-204.9) | 231 | 156.6  (138.2-179.8) | 235 | 149.3  (131.9-171.6) | 262 | 148.0  (132.4-165.7) | 278 | 139.7  (125.9-155.4) | 241 | 113.2  (101.3-126.4) |
| *Acute respiratory diseases* | 56 | 54.8  (40.4-75.0) | 54 | 60.8  (45.2-82.5) | 67 | 58.1  (44.4-123.1) | 60 | 49.4  (37.5-67.0) | 44 | 34.5  (25.5-49.1) | 53 | 36.3  (27.6-50.7) | 45 | 25.3  (19.0-33.9) | 37 | 20.0  (14.7-27.3) | 30 | 15.4  (11.0-21.2) |
| *Chronic respiratory diseases* | 171 | 158.4  (135.9-186.1) | 141 | 128.0  (107.6-154.1) | 193 | 148.3  (128.3-214.3) | 153 | 103.4  (88.4-123.3) | 141 | 93.3  (79.3-112.3) | 131 | 81.4  (68.7-99.2) | 160 | 92.0  (79.7-106.5) | 180 | 90.1  (79.0-103.0) | 174 | 81.5  (71.4-92.8) |

(b) Men-Apulia Region

| **All ages** | **1980-1982** | | **1983-1985** | | **1986-1988** | | **1989-1991** | | **1992-1994** | | **1995-1997** | | **1998-2000** | | **2001-2003** | | **2006-2008** | |
| --- | --- | --- | --- | --- | --- | --- | --- | --- | --- | --- | --- | --- | --- | --- | --- | --- | --- | --- |
|  | **No.** | **SDR**  **IC 90%** | **No.** | **SDR**  **IC 90%** | **No.** | **SDR**  **IC 90%** | **No.** | **SDR**  **IC 90%** | **No.** | **SDR**  **IC 90%** | **No.** | **SDR**  **IC 90%** | **No.** | **SDR**  **IC 90%** | **No.** | **SDR**  **IC 90%** | **No.** | **SDR**  **IC 90%** |
| Overall mortality | 46768 | 1979.8  (1938.5-2025.7) | 47102 | 2167.5  (2082.5-2262.3) | 47425 | 1810.7  (1776.3-1848.4) | 47402 | 1520.6  (1509.6-1532.1) | 49188 | 1434.7  (1423.9-1445.6) | 49602 | 1356.8  (1346.8-1366.9) | 50213 | 1303.1  (1293.8-1312.5) | 50034 | 1214.0  (1205.4-1222.7) | 51208 | 1091.7  (1084.1-1099.5) |
| All cancers | 9990 | 326.1  (318.5-339.6) | 11049 | 357.3  (348.0-380.1) | 12288 | 374.4  (366.9-386.0) | 13003 | 365.6  (360.0-371.8) | 14257 | 376.3  (370.9-381.9) | 14793 | 371.6  (366.4-376.9) | 15544 | 373.6  (368.6-378.7) | 16096 | 366.3  (361.5-371.2) | 17179 | 348.4  (344.0-352.8) |
| *Lung cancer* | 3290 | 100.6  (97.6-111.2) | 3649 | 109.6  (106.3-129.8) | 3947 | 111.5  (108.4-119.8) | 4240 | 113.6  (110.6-117.0) | 4336 | 109.8  (106.9-112.7) | 4415 | 106.3  (103.6-109.1) | 4619 | 107.1  (104.5-109.8) | 4487 | 98.7  (96.3-101.2) | 4589 | 91.0  (88.8-93.3) |
| Circulatory diseases | 19679 | 954.4  (925.8-987.7) | 19108 | 1083.2  (1014.6-1162.2) | 18347 | 810.3  (785.6-838.5) | 17622 | 647.5  (639.3-656.2) | 18211 | 592.6  (585.1-600.3) | 18391 | 549.3  (542.5-556.3) | 18422 | 515.4  (509.1-521.7) | 18079 | 466.8  (461.1-472.5) | 17141 | 383.0  (378.2-387.8) |
| *Ischaemic heart diseases* | 6617 | 277.6  (271.3-290.1) | 6271 | 268.8  (244.2-304.6) | 5918 | 213.3  (206.9-224.0) | 5919 | 188.3  (183.7-193.3) | 6044 | 176.5  (172.5-180.6) | 6054 | 166.6  (162.9-170.4) | 5927 | 153.9  (150.5-157.4) | 6266 | 154.2  (151.0-157.6) | 6064 | 132.0  (129.1-134.8) |
| Respiratory diseases | 5317 | 282.2  (260.6-309.0) | 5126 | 248.8  (227.2-281.8) | 5153 | 221.2  (215.6-231.2) | 4657 | 172.7  (167.9-177.9) | 4533 | 151.2  (147.2-155.3) | 4539 | 140.6  (137.0-144.3) | 4702 | 135.3  (132.0-138.7) | 4542 | 120.0  (117.0-123.0) | 4451 | 100.5  (97.9-103.0) |
| *Acute respiratory diseases* | 1095 | 72.1  (58.7-91.7) | 835 | 55.6  (40.3-70.9) | 675 | 35.2  (30.2-45.1) | 590 | 25.1  (23.0-27.8) | 526 | 19.3  (17.8-21.0) | 583 | 19.6  (18.2-21.2) | 656 | 19.7  (18.4-21.1) | 514 | 13.8  (12.7-14.9) | 416 | 9.6  (8.8-10.5) |
| *Chronic respiratory diseases* | 3657 | 169.4  (162.5-182.6) | 3557 | 163.9  (155.3-186.5) | 3492 | 147.3  (140.9-158.0) | 3162 | 116.4  (112.5-120.7) | 3208 | 106.8  (103.5-110.3) | 2993 | 92.6  (89.7-95.6) | 2986 | 86.2  (83.5-88.9) | 3037 | 81.0  (78.6-83.5) | 3237 | 73.6  (71.5-75.9) |

(c) Men-Italy

| **All ages** | **1980-1982** | | | **1983-1985** | | | **1986-1988** | | | **1989-1991** | | | **1992-1994** | | | **1995-1997** | | | **1998-2000** | | | **2001-2003** | | | **2006-2008** | | |
| --- | --- | --- | --- | --- | --- | --- | --- | --- | --- | --- | --- | --- | --- | --- | --- | --- | --- | --- | --- | --- | --- | --- | --- | --- | --- | --- | --- |
|  | **No.** | **SDR**  **IC 90%** | | **No.** | **SDR**  **IC 90%** | | **No.** | **SDR**  **IC 90%** | | **No.** | **SDR**  **IC 90%** | | **No.** | **SDR**  **IC 90%** | | **No.** | **SDR**  **IC 90%** | | **No.** | **SDR**  **IC 90%** | | **No.** | **SDR**  **IC 90%** | | **No.** | **SDR**  **IC 90%** | |
| Overall mortality | 861430 | 1989.9  (1985.1-1994.7) | | 860373 | 1944.9  (1940.2-1949.5) | | 840794 | 1772.8  (1771.6-1773.9) | | 843450 | 1620.0  (1617.0-1622.9) | | 849657 | 1518.2  (1515.5-1520.9) | | 850448 | 1433.6  (1431.1-1436.1) | | 844859 | 1357.0  (1354.7-1359.4) | | 839433 | 1270.7  (1268.5-1272.9) | | 833461 | 1114.3  (1112.4-1116.3) | |
| All cancers | 220200 | 418.8  (417.0-420.6) | | 234042 | 436.5  (434.7-438.3) | | 249150 | 445.6  (443.9-447.3) | | 260107 | 442.1  (440.6-443.7) | | 269332 | 437.7  (436.3-439.2) | | 271085 | 421.0  (419.6-422.4) | | 273951 | 407.8  (406.5-409.1) | | 282122 | 400.2  (399.0-401.5) | | 290215 | 369.8  (368.7-371.0) | |
| *Lung cancer* | 63262 | 109.9  (109.1-110.7) | | 68554 | 117.6  (116.8-118.5) | | 73340 | 121.8  (121.0-122.6) | | 75731 | 121.2  (120.5-122.0) | | 77700 | 119.7  (119.0-120.5) | | 77091 | 114.6  (113.9-115.3) | | 76653 | 109.6  (109.0-110.3) | | 77046 | 105.6  (104.9-106.2) | | 76047 | 94.8  (94.2-95.4) | |
| Circulatory diseases | 370120 | 963.6  (961.7-965.5) | | 360766 | 916.6  (915.4-917.8) | | 336504 | 791.8  (789.6-794.0) | | 327352 | 695.7  (693.5-697.8) | | 329995 | 643.1  (641.1-645.0) | | 329977 | 598.6  (596.8-600.3) | | 327246 | 559.6  (557.9-561.2) | | 314262 | 502.2  (500.7-503.7) | | 290879 | 405.0  (403.8-406.3) | |
| *Ischaemic heart diseases* | 130488 | 296.1  (294.3-297.9) | | 125498 | 272.7  (271.0-274.4) | | 118639 | 238.6  (237.2-240.0) | | 120333 | 227.3  (226.1-228.5) | | 120977 | 214.4  (213.4-215.5) | | 122003 | 206.7  (205.7-207.7) | | 118443 | 191.6  (190.7-192.6) | | 117450 | 180.4  (179.6-181.3) | | 112306 | 152.7  (151.9-153.4) | |
| Respiratory diseases | 69701 | 185.3  (183.8-187.0) | | 69464 | 181.4  (179.8-183.0) | | 65806 | 161.3  (160.0-162.7) | | 63046 | 139.2  (138.2-140.3) | | 59926 | 121.0  (120.1-121.9) | | 61426 | 115.6  (114.8-116.4) | | 65611 | 116.0  (115.2-116.8) | | 63174 | 103.7  (103.0-104.4) | | 62749 | 88.7  (88.2-89.3) | |
| *Acute respiratory diseases* | 19606 | 62.5  (61.3-63.7) | | 16441 | 52.1  (51.0-53.2) | | 13929 | 41.1  (40.3-42.0) | | 11625 | 29.9  (29.4-30.5) | | 10388 | 23.4  (23.0-23.8) | | 12028 | 24.5  (24.1-24.9) | | 15241 | 28.5  (28.1-28.9) | | 13135 | 22.5  (22.2-22.8) | | 10733 | 15.7  (15.4-16.0) | |
| *Chronic respiratory diseases* | 37666 | 94.1  (93.1-95.3) | | 38006 | 94.6  (93.5-95.8) | | 36306 | 85.1  (84.2-86.1) | | 37565 | 80.8  (80.0-81.6) | | 36356 | 72.5  (71.9-73.2) | | 34688 | 64.7  (64.1-65.3) | | 35545 | 62.3  (61.7-62.8) | | 35847 | 58.8  (58.2-59.3) | | 37799 | 53.5  (53.1-54.0) | |
|  |  |  |  |  |  |  |  |  |  |  |  |  |  |  |  |  |  |  |  |  |  |  |  |  |  |  |  |
|  |  |  |  |  |  |  |  |  |  |  |  |  |  |  |  |  |  |  |  |  |  |  |  |  |  |  |  |
|  |  |  |  |  |  |  |  |  |  |  |  |  |  |  |  |  |  |  |  |  |  |  |  |  |  |  |  |

Table S3: Trends in mortality from selected causes of death in the NPCS of Taranto, in Apulia Region and in Italy. 1980-2008 (2004-2005 data not available). All ages. Women. Number of deaths and standardized (Italian Census 2001) rates per 100.000.

(a) Women-Taranto

| **All ages** | **1980-1982** | | | **1983-1985** | | | **1986-1988** | | | **1989-1991** | | | **1992-1994** | | | **1995-1997** | | | **1998-2000** | | | **2001-2003** | | | **2006-2008** | | |
| --- | --- | --- | --- | --- | --- | --- | --- | --- | --- | --- | --- | --- | --- | --- | --- | --- | --- | --- | --- | --- | --- | --- | --- | --- | --- | --- | --- |
|  | **No.** | **SDR**  **IC 90%** | | **No.** | **SDR**  **IC 90%** | | **No.** | **SDR**  **IC 90%** | | **No.** | **SDR**  **IC 90%** | | **No.** | **SDR**  **IC 90%** | | **No.** | **SDR**  **IC 90%** | | **No.** | **SDR**  **IC 90%** | | **No.** | **SDR**  **IC 90%** | | **No.** | **SDR**  **IC 90%** | |
| Overall mortality | 2323 | 1259.2  (1217.9-1303.2) | | 2383 | 1236.8  (1196-1280.5) | | 2376 | 1186.1  (1147.9-1226.3) | | 2292 | 1029.2  (994.9-1064.9) | | 2417 | 957.6  (926.4-989.8) | | 2481 | 899.1  (870.6-928.5) | | 2855 | 958.2  (930.2-986.9) | | 2667 | 806.6  (782-831.8) | | 2952 | 785.6  (762.7-809.1) | |
| All cancers | 430 | 188.5  (173.1-206.8) | | 491 | 207.9  (192.3-226.7) | | 498 | 211.4  (195.4-229.5) | | 513 | 198  (183.5-214) | | 553 | 198.7  (184.8-213.7) | | 551 | 186.4  (173.5-200.4) | | 710 | 227.2  (213.4-241.8) | | 671 | 200.1  (187.6-213.4) | | 759 | 208.7  (196.3-221.7) | |
| *Lung cancer* | 19 | 8.9  (5.7-16.1) | | 20 | 13.9  (10-22) | | 21 | 14.8  (10.7-21.7) | | 22 | 15.4  (11.5-20.9) | | 23 | 16.1  (12.1-21.2) | | 24 | 12.7  (9.4-17) | | 25 | 17.8  (14.1-22.4) | | 26 | 12.5  (9.5-16.3) | | 27 | 15.8  (12.5-19.8) | |
| Circulatory diseases | 1156 | 697.9  (665-733.8) | | 1094 | 641.9  (610.7-676.1) | | 1017 | 557.6  (529.2-588.1) | | 953 | 472.2  (447.6-498.3) | | 1080 | 454.3  (432-477.6) | | 1101 | 416.1  (396.1-437.2) | | 1266 | 436.1  (416.8-456.3) | | 1132 | 344  (327.7-360.9) | | 1179 | 308.8  (294.4-323.9) | |
| *Ischaemic heart diseases* | 294 | 168.7  (152.2-188.2) | | 281 | 161.8  (145.7-181.3) | | 238 | 124.2  (110.7-140) | | 254 | 119.1  (106.7-133) | | 305 | 125.4  (113.7-138.3) | | 308 | 113.4  (102.9-124.9) | | 360 | 122.4  (112-133.6) | | 353 | 106.8  (97.7-116.7) | | 378 | 98  (89.8-106.8) | |
| Respiratory diseases | 142 | 88.5  (76.0-104.4) | | 136 | 78.7  (67.4-93.5) | | 158 | 90.6  (78.5-105.2) | | 158 | 77.6  (67.4-89.4) | | 142 | 59.1  (51.0-68.4) | | 136 | 52.6  (45.3-61.0) | | 175 | 60.3  (53.0-68.4) | | 152 | 45.9  (40.0-52.7) | | 148 | 39.9  (34.6-46.0) | |
| *Acute respiratory diseases* | 64 | 44  (34.8-56.6) | | 72 | 44.2  (35.6-56.6) | | 49 | 31.7  (24.2-41.9) | | 51 | 26.3  (20.3-34.1) | | 46 | 19.4  (14.8-25.2) | | 40 | 16.3  (12.2-21.4) | | 61 | 21  (16.8-26.1) | | 50 | 15.2  (11.8-19.3) | | 30 | 7.4  (5.3-10.3) | |
| *Chronic respiratory diseases* | 63 | 36.2  (28.6-47.2) | | 38 | 21.1  (15.6-30.6) | | 69 | 37.5  (30-47.6) | | 78 | 37.9  (30.9-46.5) | | 63 | 26.3  (21-32.8) | | 48 | 18.9  (14.6-24.3) | | 71 | 24.6  (20-30.1) | | 62 | 18.9  (15.1-23.4) | | 88 | 22.6  (18.8-27.2) | |
|  |  |  |  |  |  |  |  |  |  |  |  |  |  |  |  |  |  |  |  |  |  |  |  |  |  |  |  |
|  |  |  |  |  |  |  |  |  |  |  |  |  |  |  |  |  |  |  |  |  |  |  |  |  |  |  |  |

(b) Women-Apulia Region

| **All ages** | **1980-1982** | | **1983-1985** | | **1986-1988** | | **1989-1991** | | **1992-1994** | | **1995-1997** | | **1998-2000** | | **2001-2003** | | **2006-2008** | |
| --- | --- | --- | --- | --- | --- | --- | --- | --- | --- | --- | --- | --- | --- | --- | --- | --- | --- | --- |
|  | **No.** | **SDR**  **IC 90%** | **No.** | **SDR**  **IC 90%** | **No.** | **SDR**  **IC 90%** | **No.** | **SDR**  **IC 90%** | **No.** | **SDR**  **IC 90%** | **No.** | **SDR**  **IC 90%** | **No.** | **SDR**  **IC 90%** | **No.** | **SDR**  **IC 90%** | **No.** | **SDR**  **IC 90%** |
| Overall mortality | 42659 | 1310.8  (1297.7-1324.5) | 42540 | 1268.9  (1256.3-1282.1) | 42865 | 1180.5  (1175.8-1185.2) | 42178 | 1026.6  (1018.8-1034.5) | 44349 | 962.5  (955.2-969.8) | 45203 | 892.3  (885.7-899) | 47108 | 852.7  (846.5-858.9) | 48005 | 783.4  (777.8-789.1) | 50593 | 702.5  (697.5-707.5) |
| All cancers | 7004 | 176.6  (172.9-180.9) | 7530 | 184.5  (180.7-188.8) | 8216 | 190.5  (186.9-194.3) | 8860 | 192.8  (189.4-196.4) | 9250 | 186.8  (183.6-190.1) | 9814 | 186.6  (183.5-189.7) | 10217 | 182.3  (179.4-185.3) | 11134 | 185.1  (182.2-188) | 11903 | 176.5  (173.8-179.2) |
| *Lung cancer* | 344 | 8.8  (8-10.6) | 395 | 9.7  (8.9-11.4) | 417 | 9.8  (9-11) | 483 | 10.6  (9.8-11.5) | 470 | 9.5  (8.8-10.3) | 530 | 10.1  (9.4-10.9) | 637 | 11.4  (10.6-12.1) | 662 | 11.1  (10.4-11.8) | 849 | 12.7  (12-13.5) |
| Circulatory diseases | 22892 | 759.6  (749.4-770.4) | 21961 | 706.5  (696.7-716.8) | 21240 | 627  (621.6-632.6) | 20088 | 518.4  (512.5-524.4) | 21593 | 487.8  (482.4-493.2) | 21665 | 437.3  (432.5-442.1) | 22384 | 409.2  (404.9-413.6) | 21843 | 353.9  (350-357.7) | 21659 | 291.3  (288.1-294.6) |
| *Ischaemic heart diseases* | 5622 | 182.7  (178.2-187.9) | 4965 | 152.9  (148.9-157.5) | 4496 | 127.2  (123.8-130.9) | 4495 | 111.4  (108.6-114.3) | 4814 | 106.2  (103.7-108.8) | 5146 | 102.4  (100-104.8) | 5190 | 94.4  (92.3-96.6) | 5610 | 91.1  (89.2-93.2) | 6022 | 81.5  (79.7-83.2) |
| Respiratory diseases | 2967 | 99.1  (95.5-103.4) | 2771 | 93.6  (90.4-97.4) | 2828 | 86.1  (83.1-89.4) | 2370 | 62.1  (60-64.4) | 2259 | 50.8  (49-52.6) | 2415 | 49  (47.3-50.7) | 2842 | 52  (50.4-53.6) | 2662 | 43.1  (41.7-44.5) | 2759 | 37.0  (35.9-38.2) |
| *Acute respiratory diseases* | 1093 | 38.8  (36.2-42.3) | 925 | 34.7  (32.2-37.9) | 788 | 25.9  (24.1-28.1) | 555 | 15.4  (14.3-16.7) | 566 | 13.1  (12.1-14) | 596 | 12.2  (11.4-13.1) | 820 | 15.1  (14.2-16) | 629 | 10.1  (9.5-10.8) | 527 | 6.9  (6.4-7.4) |
| *Chronic respiratory diseases* | 1538 | 49  (46.8-52.1) | 1407 | 44.5  (42.1-47.5) | 1400 | 41.7  (39.7-44.1) | 1239 | 32.3  (30.7-34) | 1170 | 26.3  (25-27.6) | 1148 | 23.2  (22.1-24.4) | 1273 | 23.3  (22.2-24.4) | 1294 | 20.9  (20-21.9) | 1542 | 20.7  (19.8-21.6) |

|  |  |  |
| --- | --- | --- |

(c) Women-Italy

| **All ages** | **1980-1982** | | **1983-1985** | | **1986-1988** | | **1989-1991** | | **1992-1994** | | **1995-1997** | | **1998-2000** | | **2001-2003** | | **2006-2008** | |
| --- | --- | --- | --- | --- | --- | --- | --- | --- | --- | --- | --- | --- | --- | --- | --- | --- | --- | --- |
|  | **No.** | **SDR**  **IC 90%** | **No.** | **SDR**  **IC 90%** | **No.** | **SDR**  **IC 90%** | **No.** | **SDR**  **IC 90%** | **No.** | **SDR**  **IC 90%** | **No.** | **SDR**  **IC 90%** | **No.** | **SDR**  **IC 90%** | **No.** | **SDR**  **IC 90%** | **No.** | **SDR**  **IC 90%** |
| Overall mortality | 765661 | 1238.6  (1236.4-1240.7) | 779481 | 1205.8  (1203.8-1207.9) | 769365 | 1094.2  (1092.3-1096.2) | 780354 | 1007.8  (1005.9-1009.6) | 798038 | 940.9  (939.2-942.6) | 814221 | 878.9  (877.4-880.5) | 834868 | 832.3  (830.8-833.7) | 852879 | 775  (773.6-776.3) | 867305 | 684.7  (683.5-685.9) |
| All cancers | 154389 | 217.3  (216.3-218.2) | 164342 | 225.1  (224.1-226) | 173856 | 226.2  (225.3-227.1) | 183711 | 225.9  (225-226.8) | 191654 | 222.9  (222.1-223.8) | 195144 | 214.2  (213.4-215.1) | 198608 | 207.1  (206.4-207.9) | 209688 | 205.6  (204.9-206.4) | 220451 | 195.9  (195.2-196.6) |
| *Lung cancer* | 9413 | 13  (12.7-13.2) | 10669 | 14.3  (14.1-14.5) | 12137 | 15.6  (15.4-15.8) | 13227 | 16.2  (16-16.4) | 14680 | 17.1  (16.9-17.3) | 15863 | 17.5  (17.3-17.8) | 17018 | 18  (17.7-18.2) | 19040 | 19.1  (18.9-19.3) | 22392 | 20.6  (20.4-20.8) |
| Circulatory diseases | 402198 | 683.4  (681.6-685.2) | 400494 | 646.6  (645-648.3) | 382003 | 562.2  (560.7-563.7) | 378044 | 498  (496.7-499.3) | 388710 | 460.5  (459.3-461.7) | 393704 | 420.6  (419.5-421.7) | 402934 | 392.3  (391.3-393.3) | 395659 | 345.1  (344.2-346) | 377331 | 279.1  (278.3-279.8) |
| *Ischaemic heart diseases* | 99351 | 164.5  (163.6-165.5) | 94977 | 148.2  (147.4-149) | 88377 | 125.1  (124.4-125.8) | 93709 | 120.6  (119.9-121.2) | 97601 | 114.4  (113.8-115) | 103643 | 110.6  (110-111.2) | 106210 | 104.1  (103.5-104.6) | 110276 | 97.2  (96.7-97.6) | 110548 | 82.6  (82.2-83.1) |
| Respiratory diseases | 43692 | 76.3  (75.6-77) | 42588 | 70.5  (69.9-71.2) | 41070 | 61.9  (61.4-62.4) | 39449 | 52.9  (52.4-53.3) | 38118 | 45.5  (45.1-45.9) | 39770 | 42.7  (42.4-43.1) | 47237 | 46  (45.7-46.4) | 46870 | 40.9  (40.6-41.2) | 48241 | 35.8  (35.6-36.1) |
| *Acute respiratory diseases* | 20953 | 39.1  (38.6-39.6) | 17839 | 31.6  (31.1-32) | 15735 | 25.1  (24.8-25.5) | 13502 | 19  (18.7-19.3) | 12420 | 15.2  (15-15.4) | 13281 | 14.3  (14.1-14.5) | 17591 | 17  (16.8-17.2) | 15590 | 13.2  (13.1-13.4) | 12752 | 9  (8.9-9.1) |
| *Chronic respiratory diseases* | 15744 | 25.8  (25.4-26.2) | 16109 | 25.5  (25.1-25.9) | 15316 | 22.3  (22-22.6) | 16654 | 21.7  (21.5-22) | 16448 | 19.4  (19.2-19.7) | 15933 | 17  (16.8-17.2) | 17994 | 17.6  (17.4-17.8) | 19185 | 16.8  (16.6-17) | 22465 | 16.8  (16.6-17) |

Table S4: Trends in mortality from selected causes of death in the NPCS of Taranto, in Apulia Region and in Italy. 1980-2008 (2004-2005 data not available). 0 years. Number of deaths and standardized (Italian Census 2001) rates per 100.000.

(a) Infant-Taranto

| **0 years** | **1980-1982** | | **1983-1985** | | **1986-1988** | | **1989-1991** | | **1992-1994** | | **1995-1997** | | **1998-2000** | | **2001-2003** | | **2006-2008** | |
| --- | --- | --- | --- | --- | --- | --- | --- | --- | --- | --- | --- | --- | --- | --- | --- | --- | --- | --- |
|  | **No.** | **SDR**  **IC 90%** | **No.** | **SDR**  **IC 90%** | **No.** | **SDR**  **IC 90%** | **No.** | **SDR**  **IC 90%** | **No.** | **SDR**  **IC 90%** | **No.** | **SDR**  **IC 90%** | **No.** | **SDR**  **IC 90%** | **No.** | **SDR**  **IC 90%** | **No.** | **SDR**  **IC 90%** |
| Overall mortality | 162 | 364.6  (314.3-427.4) | 149 | 354.8  (303.9-419.4) | 115 | 286.0  (241.3-345.6) | 110 | 313.5  (260.1-383.4) | 104 | 313.5  (257.7-387.4) | 61 | 203.3  (156.3-269.4) | 53 | 223.7  (165.8-304.8) | 37 | 161.2  (117.8-230.3) | 28 | 99.0  (70. 5-154.0) |

(b) Infant-Apulia Region

| **0 years** | **1980-1982** | | **1983-1985** | | **1986-1988** | | **1989-1991** | | **1992-1994** | | **1995-1997** | | **1998-2000** | | **2001-2003** | | **2006-2008** | |
| --- | --- | --- | --- | --- | --- | --- | --- | --- | --- | --- | --- | --- | --- | --- | --- | --- | --- | --- |
|  | **No.** | **SDR**  **IC 90%** | **No.** | **SDR**  **IC 90%** | **No.** | **SDR**  **IC 90%** | **No.** | **SDR**  **IC 90%** | **No.** | **SDR**  **IC 90%** | **No.** | **SDR**  **IC 90%** | **No.** | **SDR**  **IC 90%** | **No.** | **SDR**  **IC 90%** | **No.** | **SDR**  **IC 90%** |
| Overall mortality | 3065 | 421.7  (407.6-436.4) | 2328 | 325.1  (312.6-338.3) | 1813 | 271.2  (259.4-283.8) | 1547 | 243.1  (231.5-255.5) | 1258 | 205.1  (194.3-216.8) | 976 | 178.6  (167.7-190.4) | 824 | 149.3  (139.7-159.9) | 694 | 134.9  (125.4-145.4) | 461 | 93.0  (85.1-101.9) |

(c) Infant-Italy

| **0 years** | **1980-1982** | | **1983-1985** | | **1986-1988** | | **1989-1991** | | **1992-1994** | | **1995-1997** | | **1998-2000** | | **2001-2003** | | **2006-2008** | |
| --- | --- | --- | --- | --- | --- | --- | --- | --- | --- | --- | --- | --- | --- | --- | --- | --- | --- | --- |
|  | **No.** | **SDR**  **IC 90%** | **No.** | **SDR**  **IC 90%** | **No.** | **SDR**  **IC 90%** | **No.** | **SDR**  **IC 90%** | **No.** | **SDR**  **IC 90%** | **No.** | **SDR**  **IC 90%** | **No.** | **SDR**  **IC 90%** | **No.** | **SDR**  **IC 90%** | **No.** | **SDR**  **IC 90%** |
| Overall mortality | 27333 | 349.0  (345.1-353.0) | 21112 | 272.4  (268.9-275.9) | 17061 | 235.9  (232.6-239.4) | 14678 | 204.6  (201.5-207.9) | 12435 | 180.4  (177.3-183.5) | 9930 | 151.7  (148.7-154.7) | 8147 | 121.1  (118.6-123.7) | 6983 | 104.6  (102.2-107.1) | 6037 | 86.2  (84.1-88.4) |
